# Design and Implementation of Practical Bidirectional Texture Function Measurement Devices Focusing on the Developments at the University of Bonn

**DOI:** 10.3390/s140507753

**Published:** 2014-04-28

**Authors:** Christopher Schwartz, Ralf Sarlette, Michael Weinmann, Martin Rump, Reinhard Klein

**Affiliations:** 1 Institut für Informatik II, Universität Bonn, Friedrich-Ebert-Allee 144, 53113 Bonn, Germany; E-Mails: sarlette@cs.uni-bonn.de (R.S.); mw@cs.uni-bonn.de (M.W.); rk@cs.uni-bonn.de (R.K.); 2 X-Rite Deutschland GmbH, Adenauerallee 170, 53113 Bonn, Germany; E-Mail: MRump@xrite.com

**Keywords:** bidirectional texture function, BTF, bidirectional reflectance distribution functions, BRDF, light field, acquisition device, high dynamic range, HDR, camera calibration, gonioreflectometer

## Abstract

Understanding as well as realistic reproduction of the appearance of materials play an important role in computer graphics, computer vision and industry. They enable applications such as digital material design, virtual prototyping and faithful virtual surrogates for entertainment, marketing, education or cultural heritage documentation. A particularly fruitful way to obtain the digital appearance is the acquisition of reflectance from real-world material samples. Therefore, a great variety of devices to perform this task has been proposed. In this work, we investigate their practical usefulness. We first idey a set of necessary attributes and establish a general categorization of different designs that have been realized. Subsequently, we provide an in-depth discussion of three particular implementations by our work group, demonstrating advantages and disadvantages of different system designs with respect to the previously established attributes. Finally, we survey the existing literature to compare our implementation with related approaches.

## 1. Introduction

The optical appearance of materials is an important stimulus for the human perception. It influences the overall impression of an object and even invokes emotions. For instance, casings made from brushed metals appear more valuable than casings made from plastics, furniture made from wood is perceived as warm and cozy, and cloth that has a silky appearance is perceived to be cooler and smoother than cloth made from wool fabrics. These effects are well known [1] and are for instance utilized in industrial product design. The ability to have these associations is deeply rooted in our nature. In the case of foods, we are used to gauge the freshness based on the appearance of the surface. For human skin, we are even able to see subtlest differences and assess things such as healthiness or mood of our fellow men.

For our application, we define optical *appearance* by the impression of a scene when perceived by an observer, e.g., on the retina or a camera sensor. In the simplest case, it can be reproduced by presenting a photographic image. However, the impression can change—sometimes drastically—depending on various inherent and external factors. Among those are the geometry and spatial variation of optical properties of an object as well as aspects related to the observation, such as illumination and point of view. Human perception is trained to assess appearance of materials in combination with the given environmental factors. It is therefore necessary to explicitly consider this dependency. One possible scenario is the usage of computer graphics to generate images that convey the correct impression for a given set of conditions.

Faithful digital material appearance reproduction is a prerequisite for special effects as well as sensible computer based product design and virtual prototyping. Digital material models can even help to aid real-world production processes by providing a well-defined specification of the desired appearance. They can also be used for the creation of virtual surrogates of real-world objects, e.g., for display of fragile or precious cultural heritage aacts or products in online shops. Yet, good digital optical material descriptions are mandatory for the generation of plausible, photo-realistic, or even predictive rendered images that convey the desired appearance.

A colorful bouquet of mathematical models to characterize the appearance of materials in dependence of illumination and observation conditions exists, distinguishing between different levels of complexity of the optical phenomena that can be described. In this work, we will focus on the *Bidirectional Texture Function* (BTF) [2], a high-quality and very general model for digital material appearance. The BTF is capable of reproducing the appearance of a material in dependence of the illumination direction, the viewing direction and the spatial position on the object surface. It is able to account for optical effects that originate from complex or intricate surface structures, such as fur, cracks, bumps or weaving patterns, without the need to explicitly model them. To some extent, sub-surface light transport is captured as well. This way, the realistic impressions of a large variety of materials encountered in everyday life can be achieved. The compression, transmission, editing, rendering and automatic classification of BTFs have thus been active areas of research in the fields of computer graphics and computer vision for more than a decade.

The key to the realistic impression is the data-driven nature. BTFs are usually generated by systematically tabulating the *reflectance* of real-world samples. The reflectance, *i.e.*, the amount of electromagnetic power that is reflected by the surface, can vary depending on the same parameters as the appearance model. For being truly general, brute force approaches, densely sampling all dimensions of the parameter domain, need to be considered. This way, the BTF can directly be used in optical simulations to enable the faithful reproduction of the material appearance. Furthermore, densely sampled reflectance data can serve as a basis for the development and evaluation of specialized light-weight acquisition devices and elaborate mathematical material descriptions. Therefore, the accurate capture of BTFs requires the thorough acquisition of billions of data points.

In this article, we will first provide the reader with the necessary background on the BTF, its physical interpretation and its relation to other scattering distribution functions in Section 2. From this, we derive general design requirements for a BTF measurement setup. By surveying the literature, we establish a categorization of existing setups in Section 3. Subsequently, we will describe the design and implementation of three exemplary setups for the image based acquisition of BTFs from materials samples in Sections 4–7, each following different premises. In doing so, we will discuss the challenges of measuring spatially varying bi-directional reflectance and the respective solutions that were found and implemented for the devices in detail. As the requirements and processes have shifted over time, all three designs have unique advantages and limitations, which will be juxtaposed for the sake of comparison. In Section 8, we will compare the implemented approaches with each other and with other setups proposed in the literature. Finally, we draw our conclusions and point out avenues for future research in Section 9.

Note that, although our three setups that we discuss in later sections have been part of previous publications, this article contains several technical details and insights that have not yet been reported.

## 2. Physical Background and Design Requirements

In order to establish design requirements for the measurement of reflectance, we briefly discuss what physical properties need to be captured and how this can be achieved. Understanding the scattering and distribution of light has a long standing tradition in science. Even in computer graphics several models exist that have different complexity and descriptiveness. For our case, we focus on light interaction that occurs at the boundary surfaces between matter and air and neglect scattering in participating media outside the object. In the most general case, the light transport within an object can then be described using the 12-dimensional *scattering function* [3]:

(1) $$S(\omega_{i},{\text{x}}_{i},\lambda_{i},t_{i};\omega_{o},{\text{x}}_{o},\lambda_{o},t_{o})$$

**Figure 1.**
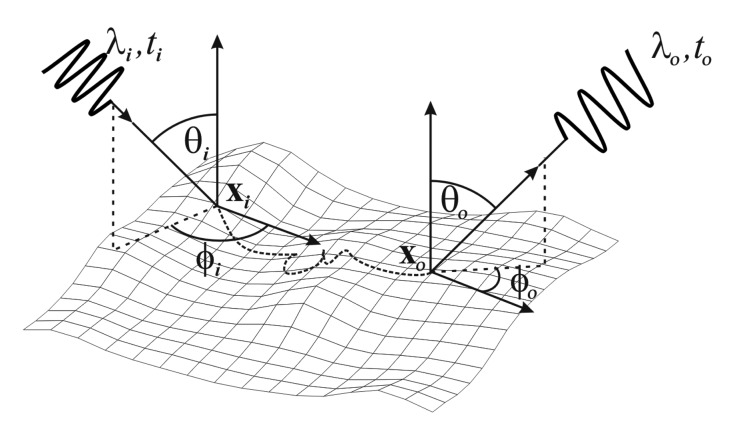
The parameters of the 12-dimensional scattering function, enabling the description of light interaction with objects bounded by a surface. Image taken from [5].

Then, *p* := *S* cos *θ_o_* d*a* d*ω* dλ d*t* denotes the probability of a photon with wavelength λ*_i_* coming from direction *ω_i_* = (*θ_i_, ϕ_i_*)*^T^* that is entering the object at surface position x*_i_* = (*x_i_,y_i_*)*^T^* at the time *t_i_* to be scattered inside the object in such a way that it leaves the surface through a surface patch of size da at position x*_o_* = (*x_o_,y_o_*)*^T^* in a time interval d*t* at time *t_o_* into a solid angle d*ω* around direction *ω_o_* = (*θ_o_, ϕ*_o_)*^T^* within wavelength range dλ containing λ*_o_*. The direction *ω_i_* is also often referred to as the illumination direction, and similarly *ω_o_* can be denoted as the viewing direction. Both are given with respect to the local surface orientation at x*_i_* and x*_o_* respectively. A schematic diagram of the parameters can be found in Figure 1.

When neglecting the distribution of radiance over wavelengths and time, the scattering can be modeled using the 8D *Bidirectional Scattering-Surface Reflectance Distribution Function* (BSSRDF) [4]:

(2) $$S(\omega_{i},{\text{x}}_{i};\omega_{o},{\text{x}}_{o})$$

In practical applications, however, it may often not be desirable or even possible to describe the true surface of an object. In the case of clothing, for example, the real surface, *i.e.*, the boundary between the object-matter and air, would be the surface of the individual fibers that are spun to yarn, woven to fabric and sowed to the item of clothing. Describing the appearance of clothes by the scattering of light at the level of individual fibers requires a simulation with an enormous complexity and a complete model of the surface, including every single fiber. Although for highly specialized domains such as cloth this approach is actually followed [6], in more generic applications, often an approximation of the surface must suffice. The fine details of the surface that make up the material appearance should then be captured by an appropriate reflectance model, such as the BTF.

### 2.1. Reflectance Fields

When considering a single moment in time and a fixed wavelength, the radiance for all light rays originating at a point in space **X** ∈ (x0211D;^3^ and heading into direction *ω* = (*θ, ϕ*)*^T^* is described by the *plenoptic function P*(**X**, *ω*) [7]. Taking a photographic picture, observing a scene with the naked eye or synthesizing a computer generated image all amount in sampling a 2D slice of the respective plenoptic function. This insight can be used for the generation of novel images without computing a full blown optical simulation of the light scattering on the true scene surface. Instead, the plenoptic function can be sampled from a real-world exemplar using photographs and the appearance from new viewpoints can be reconstructed from these samples.

In the absence of a participating medium or solid occluders, the radiance and thus the plenoptic function is constant along rays. Consider an arbitrarily complex surface that is encapsulated in a virtual bounding volume *V*, such that the observer is always located outside *V*. It is then sufficient to sample the radiance originating from points x ∈ *∂V* ∈ ℝ^2^ on the surface of the bounding volume into the outbound directions *ω_o_* to faithfully reconstruct the appearance for a given, static illumination [8,9]. The 5D plenoptic function *P*(**X**, *ω*) is reduced to a 4D *light field L_o_*_,_*_V_*(x,*ω_o_*) parameterized over the bounding surface of *V*. Similarly, if the observer is always inside the bounding volume, the appearance of a completely static scene on the outside is fully described by the light field *L_i_*_,_*_V_*(x, *ω_i_*) with inbound directions *ω_i_* [9].

**Figure 2.**
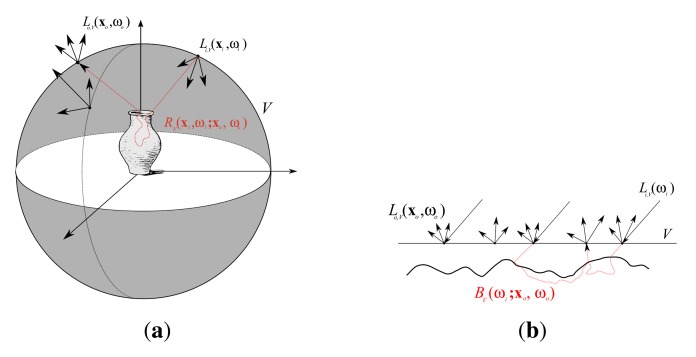
Illustration of the observation that the outgoing light field *L_o_*_,_*_V_*(x*_o_, ω_o_*) depends on the incident light field *L_i_*_,_*_V_*(x*_i_, ω_i_*), both parameterized over the surface of the bounding volume *V*. This dependency is fully described as the reflectance field *R_V_*(x*_i_, ω_i_*; x*_o_, ω_o_*), shown in (a). When considering a planar bounding surface and far-field illumination one can derive the BTF depicted in (b). Images taken from [11]. (**a**) reflectance field; (**b**) BTF.

However, light fields only sample static scenes, *i.e.*, fixed lighting, objects and materials. Varying the illumination will lead to completely different plenoptic functions. Debevec *et al.* [10] make the observation that for a given bounding volume *V* the outgoing light field *L_o_*_,_*_V_* is directly dependent on the incident light field *L_i_*_,_*_V_* (see Figure 2a). The authors use this to describe the exitant radiance from *V* under every possible incident illumination as an 8D *reflectance field*

(3) $$R_{V}(L_{i,V};L_{o,V})=R_{V}({\text{x}}_{i},\omega_{i};{\text{x}}_{o},\omega_{o})$$

Given that the observer and illumination are outside of *V*, the reflectance field can be used for reconstructing appearance under arbitrary new viewpoints and illumination conditions. For this purpose, the outgoing light fields are sampled under a set of basis incident light fields. New light variants are reconstructed as a linear combination of the illumination basis by exploiting the principle of superposition.

Note that the reflectance field is closely related to the BSSRDF *S* in Equation (2). When using the true surface as *∂V*, both functions are identical. Yet, the approximation using a boundary surface makes the reflectance field easier to sample and reconstruct, as was attempted in [10,12–15] (The setup used in [15] has previously been published in [16]). In turn, this means that reflectance fields, and thus also BTFs, are completely eligible for use in optical light scattering simulations as if they were BSSRDFs. The only restriction is that all scattering events occur outside and on the bounding volume. The light scattering within the bounding volume is already encoded in the reflectance field and does not need to be simulated.

### 2.2. Bidirectional Texture Functions

When assuming far-field illumination, *i.e.*, the sources of the illumination are always infinitely far away, the incident radiance for a given direction *ω_i_* is the same at all points x*_i_*: ${\text{x}}_{i}:L_{i,V}({\text{x}}_{i},\omega_{i})=L_{i,V}^{d}(\omega_{i})$ (see Figure 2b). This reduces the dimensionality to a 6D reflectance field that is called the *Bidirectional Texture Function* (BTF) [2]:

(4) $$B_{V}(\omega_{i};{\text{x}}_{o},\omega_{o})$$

For the purpose of representing material appearance, the proxy surface *∂V* is usually considered to be planar, as depicted in Figure 2b, but can in principle still be an arbitrary surface bounding the sample. The restriction to far-field illumination is a reasonable approximation for the application of material appearance representation. Compared to the size of the geometric details found in the true material surface, e.g., small bumps, cracks or fibers, the illumination is located several orders of magnitude away from the surface. Therefore, incident rays from one source are locally almost parallel and the spatial variation of illumination will in most cases be much lower than the spatial variation within the material.

Much like the reflectance field is related to the BSSRDF, the BTF closely resembles the *Spatially Varying Bidirectional Reflectance Distribution Function* (SVBRDF) *ρ*_x_(*ω_i_*;*ω_o_*). The SVBRDF basically assigns a separate *Bidirectional Reflectance Distribution Function* (BRDF) to each point on the surface. Yet, by definition a BRDF *ρ*(*ω_i_*; *ω_o_*) [17] implies physical properties which are only satisfied if scattering is a completely local phenomenon, *i.e.*, ∀x*_i_* ≠ x*_o_* : *S* = 0 in Equation (2). Therefore, SVBRDFs cannot account for non-local light transport, such as subsurface scattering.

When assuming only an approximate geometry *V*, materials often exhibit even more non-local appearance effects. The reflectance behavior at one point on a surface can be influenced by neighboring, material inherent structures, e.g., small fibers in wool yarn of woven fabrics, which are not considered in the proxy geometry. These structures may cast shadows or interreflections, occlude the point from certain views or transport light via subsurface scattering. Furthermore, these structures may very well be smaller than the spatial resolution that is used in the digitized representation of the material. Therefore, the orientation of the surface itself, and with it the direction-dependent reflectivity, could vary within one spatial sampling point. The outgoing light fields in the BTF by their nature include all of these subtleties. Thus, the BTF is applicable to faithfully capture the appearance of all kinds of optically dense materials that exhibit only localized subsurface scattering. High-quality results have been reported using BTFs for reproducing the appearance of many material samples [2,18–20] as well as complete objects [21–23].

### 2.3. Design Requirements of a BTF Measurement Apparatus

To enable the acquisition of BTFs, several basic attributes and abilities should be considered by the design of a measurement setup: Light Field capture, controlled illumination, high dynamic range imaging, radiometric calibration, spectral sampling and 3D scanning. Last but not least, although not strictly necessary from a theoretical standpoint, practical requirements should be considered as well. In the following, we will give a detailed explanation of each one.

**Light Field Capture:** Any setup that measures BTFs has to capture outgoing light fields *L_o_*_,_*_V_* from real-world material exemplars. As argued in [9], outgoing light fields are best sampled taking a set of photographic images 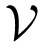 = {*I_j_*}*_j_*. of the exemplar from different camera positions on a sphere around the bounding volume, always facing the sample. For a planar bounding surface a hemisphere of positions is sufficient as positions below the plane would not be observations from outside the volume any more and hence invalid samples.

**Controlled Illumination:** To account for varying illumination, it is necessary to consider arbitrary far-field incident light fields $L_{i,V}^{d}$. As in [10], the principle of superposition can be exploited. The setup has to be capable of controlling the lighting and alternate through a set of basis illuminations 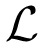 = {*L_k_*}*_k_*, from which any far-field incident light field can be reconstructed as a linear combination $L_{i,V}^{d}\approx {\text{∑}}_{k}l_{k}L_{k}$ with corresponding weights *l_k_*. It is furthermore important that the basis illumination is homogeneous over the complete sample surface in order to fulfill the far-field assumption of the BTF. In practice, most setups choose a set of approximately directional light sources, such that each basis illumination sheds light from a single direction *ω_i_*.

**High Dynamic Range Imaging:** Material reflectance usually exhibits rather high dynamic ranges. On the one hand, high radiance values are observed when the light that is reflected comes from the perfect mirroring direction. On the other hand, light from grazing angles in combination with a view direction outside the specular lobe of the material leads to very low radiance values. In spatially varying materials there can also be a considerable difference in material albedos as well as self-shadowing. This further increases the ratio between largest and lowest observable values. However, the dynamic range that can be captured by CMOS or CCD sensors of digital cameras is limited and easily exceeded by the reflected radiance. Data directly coming from the sensor is therefore usually attributed as *Low Dynamic Range* (LDR). If used directly, this either results in faulty measurements due to over-saturation of sensor pixels or—if exposure time is minimized to compensate for this effect—in extremely high noise levels in all other pixels. Thus, it is good practice to employ exposure bracketing to generate a *High Dynamic Range* (HDR) image from multiple differently exposed LDR images [24,25] to be capable of capturing the full range of reflectance values.

**Radiometric Calibration:** For the BTF to be applicable in predictive light transport simulations, any measurement setup should be carefully radiometrically calibrated. The sampled entries then give the ratio of differential incoming flux to differential reflected radiance in sr^−1^ for the given combination of directions *ω_i_* and *ω_o_*, wavelength λ and surface position x.

**Spectral Sampling:** Surface appearance is dependent on the spectrum of the light. A BTF measurement setup should at least be able to capture tristimulus images and provide for a basis illumination such that the perception of the material for a human observer under natural illumination (e.g., daylight) is captured. However, to facilitate the predictive simulation of different light sources, a dense hyper-spectral imaging of the material appearance would be preferable. Capturing reflectance of fluorescent materials would even require bi-spectral measurements.

**3D Scanning:** In case that flat real-world material samples cannot be employed for acquisition, e.g., for f naturally curved materials such as egg-shell, or if the reflectance behavior of objects or their parts should be digitized, it is useful to additionally capture the 3D shape of the probe. In principle reflectance fields could also suffice with a coarse proxy geometry, e.g., a bounding-sphere. Yet, having a more precise geometric shape model of the surface is advantageous for compression as well as rendering [22,26]. Furthermore, light simulation based on reflectance fields is only correct if the proxy geometries do not intersect each other. Too expansive bounding surfaces therefore unnecessarily limit the possible arrangements of digitized objects. BTFs introduce the additional issue of disocclusion by the proxy geometry, as light transport through transparent parts of the proxy geometry is modeled insufficiently.

A large body of work exists on the acquisition of 3D geometry, using a whole bunch of different approaches. Many off-the-shelf solutions are available, covering the full range of options in terms of accuracy as well as price. Good overviews can be found in [27–29]. However, including a 3D scanning solution into the process of reflectance capture is not a trivial task. For an automated acquisition, the 3D scanning hardware should better be integrated into the reflectance measurement device, which restricts the possible 3D acquisition approaches. If an external device is employed, the issue of registration of the 3D measurement with the reflectance samples has to be tackled.

**Practical Requirements:** It is in general not sufficient to capture only a few radiance values. For a faithful reconstruction, the sampling rate in all six dimensions of the parameter space should be adequately high (consider the Nyquist-Shannon sampling theorem [30]). For this, millions and billions of data points have to be recorded. That makes a computer-controlled setup mandatory, as manual sampling or extensive user-interaction would make the process completely infeasible. Here, the measurement time should be as short as possible and the sampling as dense as necessary. The reflectance samples should be of high quality: All spatially varying effects that are not due to the reflectance, such as sensor noise, inhomogeneities of illumination, *etc.*, should be eliminated. The actual sampling directions should show as little variation from the ideal directions as possible. For industrial application, the setup also has to function reliably without supervision and show a high durability as well as the capability to measure in rapid succession. Finally, the measurement volume should be large enough for the application, *i.e.*, capturing all the spatial variations in material samples or even complete objects.

## 3. Classification of Device Designs

By far not all reflectance acquisition setups found in literature aim to fulfil all of the above design requirements. The measurement, representation and reproduction of optical phenomena is an interdisciplinary and very active field of scieic research with a lot of specialized solutions. Excellent surveys on techniques for surface reflectance acquisition and representation are given in [5,31–34]. In this work, we will consider only those publications about setup designs that are the most relevant to our application, *i.e.*, that are in principle capable to meet the requirements established in Section 2.3.

Among those, we have ideied three general categories of BTF measurement devices: Gonioreflectometers, mirror based setups and camera array setups. Still, the individual designs often follow additional application specific approaches and differ with respect to speed, flexibility, resolution or complexity. In the next paragraphs, we provide a brief summary of the categories and the covered publications. A more detailed discussion and comparisons of the setups can be found later in the article in Sections 8.2.1–8.2.3.

### 3.1. Gonioreflectometer Setups

Classically, a gonioreflectometer is a device consisting of a light source and a photo-detector. A bi-directional reflectance measurement is performed by moving the employed light source and the detector to several different locations around the sample. Gonioreflectometers have been employed for the measurement of BRDFs for a long time [4,35–40]. In [37], for instance, a fully automated BRDF sample acquisition is presented that can assume all angular configurations on the hemisphere above the exemplar. In their setup, the light source and the detector are mounted on movable mechanical arms and the material sample and the light source arm are additionally mounted on a turntable and a ring bearing respectively. Recent publications additionally focus on hyper-spectral BRDF acquisition [35,38–40].

Gonioreflectometers can be used to acquire spatially varying reflectance by employing a spatial camera-sensor (CMOS or CCD) instead of a single photoresistor. Different ways to achieve the bi-directional measurement have been explored. Several setups propose to have the light source or the detector at a fixed position and achieve the necessary angular configurations by changing the orientation of the material sample [2,19,20,41–44]. The setups proposed in [45,46] instead move both, sensor and light source, around the sample.

We exemplary present our own gonioreflectometer setup [5,19,47,48] in detail in Section 5.

### 3.2. Mirror and Kaleidoscope Setups

For taking many BRDF measurements on the same sample in parallel, Ward *et al.* [49] proposed a setup with a half-silvered mirror in combination with a CCD fish-eye camera. Using the mirror they were capable of capturing the full hemisphere of view directions *ω_o_* simultaneously. This idea was followed in several subsequent publications, such as [50] or [51]. A projector is used to illuminate a specific point on the mirror. The ray is reflected and illuminates the sample surface from a direction *ω_i_*. The scattering of the incident light by the material sample is observed through the same mirror by a camera that has an identical optical axis as the projector by using a beam splitter.

The same principle can be applied for measuring spatially varying reflectance by moving the mirror on a translation stage to capture reflectance at different points on the surface [52–54]. Alternatively, a piecewise planar mirror geometry can be employed in order to allow a spatially extended illumination and observation of the sample under constant directions. This can either be a few mirrors arranged as a kaleidoscope [18,55], utilizing interreflections to form more directions, or an elliptical arrangement of several piecewise planar mirrors [12,13,16], showing only the direct reflection.

We do not provide an exemplary implementation for a mirror based setup. As we argue in Section 8.2.2. , this class of devices can have some considerable drawbacks with respect to accuracy, possible sample size and resolution. We therefore direct our focus on camera array setups as a more practical alternative with similar advantages.

### 3.3. Camera and Light Array Setups

Similar to kaleidoscopic setups, camera arrays feature a parallel acquisition of the spatial dimensions x and (parts of) the outgoing directions *ω_o_*. Yet, in contrast to the mirror based setups, multiple cameras are employed for the simultaneous direction acquisition, so that the full sensor resolution can be utilized for the spatial domain. Often, camera arrays are combined with light arrays, avoiding time-consuming mechanical re-positioning steps of a light source.

Existing camera array setups either consist of a few fixed cameras [10,56–63] that sample only a slice or sparse set of the possible view directions—sometimes complemented with a turntable [21,64–71] to cover a larger set of directions—or employ a dense hemispherical camera arrangement [5,23].

We will present two camera array setups that we implemented ourselves in Sections 6 and 7. We denote the setups as *Dome 1* and *Dome 2*. The first one [5,22,23,72] consequently follows the approach of simultaneous view direction acquisition, capturing the full outgoing light field at once with a large number of cameras. The second one [71,73,74] implements a semi-parallel acquisition, using fewer cameras in combination with a turntable.

### 3.4. 3D Shape Acquisition in Reflectance Measurement Devices

Examples of setups that also perform an integrated 3D shape acquisition to facilitate reflectance capture on curved surfaces can be found in all three categories.

In [21,22,64,65] a coarse shape is reconstructed from object silhouettes. The authors of [21,59,60] employ additional auxiliary 3D scanners and register the geometry to the reflectance measurement. Several other devices [23,45,55,69–71,74] instead rely on an integrated structured light approach. This holds the advantage that the geometry is already registered with the reflectance measurements.

## 4. General Implementation Considerations

In order to illustrate the possible design choices that can be followed in the different device categories, we exemplarily discuss three specific implementations. In Sections 5–7 we will provide an in-depth description of the three reflectance measurement devices that were implemented at the Institute of Computer Science II of the University of Bonn. The detailed discussion is intended to aid the interested reader and explain the reasoning behind the respective design choices. While the description of the employed hardware is rather particular, other information, e.g., the abstract design or employed calibration methods, will also provide valuable insight beyond the individual setups.

In this article, we will consider the acquisition pipeline implemented in the devices up to the point that a full tabulated BTF tensor **B** ∈ ℝ^| 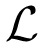
|×| 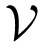
|×|Λ|×|^*^χ^*^|^ is recorded. Here, 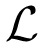, 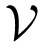 and Λ denote the sets of basis illuminations, view directions and wavelength bands. *χ* is the set of sampled spatial positions on the bounding surface *∂V*. In case of a classical BTF measurement [2] from a planar, rectangular material sample, we use a planar reference geometry. Then, *χ* = {1, 2,…,*W*} × {1, 2,…,*H*} is simply a full discrete grid with spatial resolution *W* times *H*, which can be mapped to the rectangular surface patch with an affine transformation. For BTFs on arbitrary geometries, we assume a planar embedding of the bounding surface into a finite rectangle exists. The spatial positions *χ* ⊂ {1,2,…,*W*} × {1, 2,…, *H*} are given by a bijection onto that rectangle, *i.e.*, as a texture map of resolution *W* × *H*. Note that in both cases 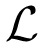 and 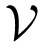 refer to the direction sampling of the measurement device. Several approaches in literature, e.g., surface light fields [26] or surface reflectance fields [10], propose to perform a resampling step and eventually store the reflectance with respect to a surface. This requires to bring samples into local directions, which are given with respect to the surface normal and tangent in each point in *χ*. While this is generally a good idea, we will not tackle the resampling of BTFs in this article but refer for example to [22,23,75,76].

As the acquisition of BTFs requires full control over the illumination conditions, all of our setups have in common that they operate in a controlled lab environment. Similar to the measurement laboratory reported by Goesele *et al.* [77], our provisions resemble those of a photographic studio. We sealed all windows with opaque black foil to avoid any outside illumination. We further blackened the ceiling, and laid a dark carpet and black curtains to minimize the effect of stray light. Finally, all parts of the employed equipment that potentially face a camera or the material sample have been painted with a diffuse black coating as well. Status-LEDs of the close-by control computers have been disconnected or blinded with black duct tape.

**Figure 3.**
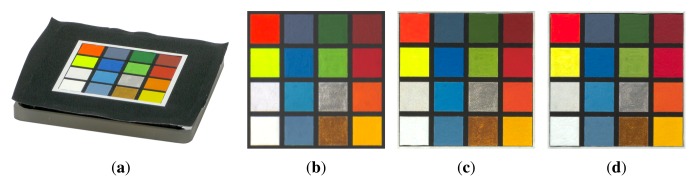
A handmade material chart (a), used for comparing the accuracy of our three setups. Fields column-wise: Fluorescent Red, Fluorescent Yellow, Paper, White, Blue3, Blue2, Blue1, Turquoise, Green2, Green1, Silver, Gold, Red2, Red1, Orange, Yellow. (b)–(d) show reied and radiometrically corrected measurement images (*θ_i_* = 45°, *ϕ_i_* = 0°, *θ_o_* = 0°, *ϕ_o_* = 0°) from each of our three setups. For illustration purposes, the result images are tonemapped and converted to sRGB colorspace. (**a**) overview picture; (**b**) gonioreflectometer; (**c**) Dome 1; (**d**) Dome 2.

Obtaining high-quality reconstructions of surface reflectance behavior imposes the precise geometric and radiometric calibration of the involved components. The geometric calibration consists of the intrinsic and extrinsic parameters of cameras, projectors and light sources with respect to the sample. The radiometric calibration of all components establishes the radiometry of light source, sensor and lens system. This includes spatially varying effects of the optics, e.g., vignetting and light fall-off, as well as colorimetry of the sensors, *i.e.*, color-profile and white-balance. All three devices show fundamentally different requirements and approaches to achieve an accurate calibration, which are described in Sections 5.2, 6.2 and 7.2. Yet, all three setups have in common that they use additional black-and-white border markers to further improve the spatial registration of the measured data. The borders are automatically detected in the raw images using contour finding and line fitting. Then we determine the corners of the corresponding quadrilateral with sub-pixel precision using the active contour model proposed by Chan and Vese [78]. The pixels within the quadrilateral, *i.e.*, the material reflectance samples, are transformed to the respective reied *W* × *H* image by computing the homography to the common planar proxy. An example is shown in Figure 3.

Some important attributes, such as dynamic range, repeatability and accuracy of the assembled system, cannot directly be determined from the individual hardware vendors' specification sheets. To allow a meaningful discussion of these attributes, we conducted a series of experiments as part of this work. The description of the experiments can be found in Section 8.1. However, the insights from the experiments are already included in Sections 5–7.

## 5. Gonioreflectometer

**Figure 4.**
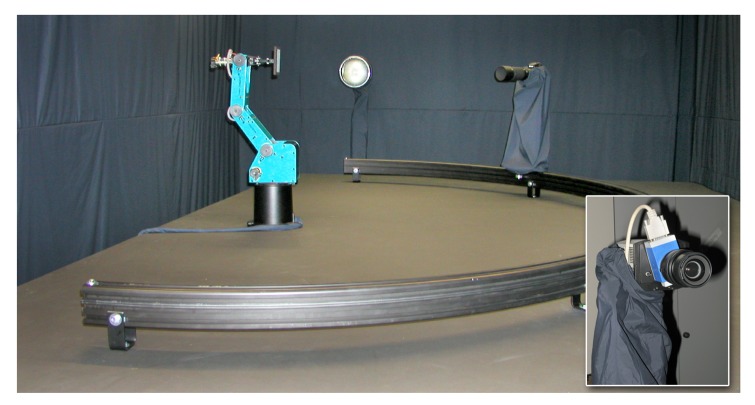
Our gonioreflectometer setup with the original equipment from 2002. Image taken from [47]. The inset on the lower right side shows the camera that is employed for hyper-spectral measurements since 2009. Note the tunable spectral filter behind the lens.

Our gonioreflectometer setup (see Figure 4), published in [5,19,47,48], was constructed between 2001 and 2002 to allow spatially varying and bi-directional measurement of material appearance from flat samples. It was extended in 2009 and is since then equipped to perform hyper-spectral measurements.

### 5.1. Hardware

The design of the device was intended to follow and improve upon the original BTF measurement approach proposed by Dana *et al.* [2]. In contrast to [2], in our setup the camera changes the position automatically, avoiding cumbersome manual placement and orientation. This is achieved via a computer-controlled rail system. The rail is bent such that the orientation of the camera towards the sample is maintained on every position. Furthermore, the robot employed by Dana *et al.* could not rotate the sample around its normal direction, *i.e.*, sample the angle *ϕ_o_*. For measuring anisotropic materials, they proposed to manually change the orientation by moving the sample and performed a second measurement. This procedure poses considerable effort but still yields only a very coarse sampling of *ϕ_o_* with two directions. In contrast, our setup employs a robot that is capable of assuming all necessary poses for an automated and dense sampling of the full angular domain. In their setup, Dana *et al.* employed a professional 3 CCD video camera with analog output together with a VGA-resolution frame-grabber. This particular combination showed a lot of color noise and only captured a single, fixed exposure in LDR with 8 *bits per pixel* (BPP). We instead utilize high-resolution digital still cameras with favorable noise characteristics and higher bit depths of 12 BPP, yielding a higher dynamic range.

#### 5.1.1. Robot & Rail

As in [2] the employed light source is placed at a fixed position. The camera however, can be moved into different azimuthal angles *ϕ_o_* via a custom built semicircle-rail-system. An Intellitek SCORBOT-ER 4u robot arm is placed in the center of the semicircle. It is used to present the mounted material sample to the camera in such a way that, in combination with the rail-system, every angular configuration (*θ_i_, ϕ_i_, θ_o_, ϕ_o_*) on the view and illumination hemispheres above the sample can be reached. Table 1 shows the measurement directions on the hemisphere above the material sample that are used. For this, the robot arm tilts and turns the sample—even in headlong positions. Unfortunately, the necessity to move the sample into slant positions makes the acquisition of 3D objects or delicate and granular materials infeasible. Rail, lamp and robot are affixed on a solid laboratory bench. Camera and light source have a distance of 170 cm and 240 cm to the material sample, respectively.

Due to constraints in the working envelope of the robot, not all azimuthal configurations for *θ_o_* > 80° can be reached reliably. To still capture direction samples for views below 80° inclination, the measurement is paused at one point and the light source is manually re-positioned at the opposite side of the rail, avoiding borderline robot poses.

#### 5.1.2. Camera

In its original configuration, reported in [19,47], a Kodak DCS760 *digital single-lens reflex* (DSLR) camera with a 6 Megapixel CCD was employed. The camera captures raw images at 12 BPP, yielding a dynamic range of 35 dB, with a Bayer-patterned *Color Filter Array* (CFA) to measure RGB color. The camera was replaced in 2004 [5] by a Kodak DCS Pro 14n with a 14Megapixel full-frame CMOS sensor to achieve higher spatial resolutions. The DCS Pro 14n also captures Bayer-patterned raw images with 12 BPP, but has a lower dynamic range of 31 dB. The choice of camera was also influenced by the fact that Kodak provided a *Software Development Kit* (SDK) that supported changing the camera settings as well as capturing raw images and directly transmitting them to a remote PC.

For performing hyper-spectral measurements [48] the setup is now equipped with a 4 Megapixel Photometric CoolSNAP K4 camera. The camera has a Peltier cooled mono-chrome CCD chip with 12 BPP, which is sensitive to electromagnetic radiation from 350 to 1, 000 nm. As the sensor is operated at approximately −25 °C, it exhibits a very low noise level despite the prolonged exposure times necessary to capture the low amount of radiance passing the narrow spectral band-filters. Thus, the cameras achieves approximately 32 dB dynamic range in a single shot. 32 different wavelength bands between 410 and 720 nm are sampled with a bandwidth of 10 nm via a CRi VariSpec multi-spectral tunable liquid crystal filter (see inset in Figure 4).

**Figure 5.**
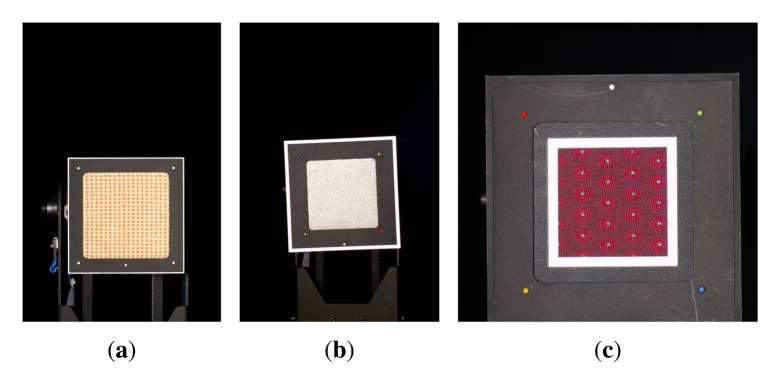
Original measurement images for *θ_o_* = 0°, taken with the Kodak DCS760 (a), the Kodak DCS Pro 14n (b) and the Photometric CoolSNAP K4 (c). The pictures also illustrate the progression in the design of the sampleholder. Notice the increase in size of the registration border and the utilization of an additional inset (c). (**a**) 2002; (**b**) 2004; (**c**) 2010.

On the two Kodak DSLRs, a Nikon AF 28–200 mm/3.5–5.6 G IF-ED lens was used at 180 mm focal length. Note that the CCD sensors of the cameras have different extents. The 35 mm equivalent focal length is therefore 240 mm for the Kodak DCS760 and 180 mm for the Kodak DCS Pro 14n. The Photometric CoolSNAP K4 is used with a Schneider-Kreuznach Componon-S 5.6/135 lens with 135 mm (35 mm equivalent of 270 mm). Figure 5 shows the field of view of the respective cameras. The maximum spatial resolution of the material sample is 280 DPI, 330 DPI and 290 DPI for the camera models.

#### 5.1.3. Light Source

**Figure 6.**
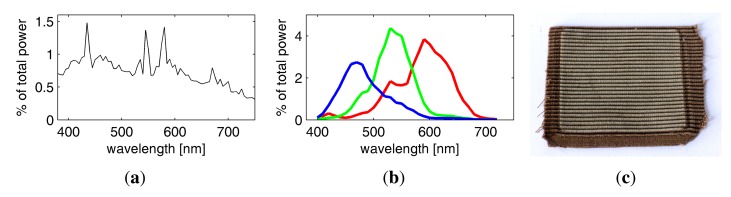
Spectral power distribution of the employed HMI bulb (a) and sensitivity of the DSLR camera (Kodak DCS Pro 14n) (b). The red, green and blue curves correspond to the respective primaries in the Bayer pattern. (c) shows the damage caused prior to installing a UV filter in front of the lamp: The uncovered area of the material sample is bleached due to prolonged UV exposure during measurement. (**a**) HMI bulb; (**b**) DSLR Camera; (**c**) UV damage.

As a light source, we employ a full-spectrum Broncolor F575 lamp with a 575W Osram *Hydrargyrum Medium Arc Length Iodide* (HMI) bulb. We use a parabolic reflector to achieve a directional light characteristic, so that the incoming directions *ω_i_* are approximately the same at every point on the sample surface. After initial experiments, a UV filter was added to prevent damage of the material sample (see Figure 6c) from the prolonged exposure of several hours necessary for full BTF measurements. Still, the lamp shows an even distribution of energy across all wavelengths considered by the RGB Bayer pattern CFAs or the spectral filter (see Figure 6) and has a color-temperature of 6, 000 K. This facilitates to capture a natural impression of the reflectance with color characteristics comparable to day-light illumination when employing the RGB sensors of the Kodak DSLRs.

We also tested an Oriel *Quartz Tungsten Halogen* (QTH) lamp with 1, 000 W and a very smooth spectrum at a color temperature of 3,200 °K. However, the lamp was disregarded because it showed a very low energy in the blue spectral bands and has an expected lifetime of 150 h, allowing only two hyper-spectral measurements in a row.

To initially determine the accurate placement of the lamp, the robot presents a planar white-target with increasing inclination angles *θ_i_*. The brightness of the target is observed through the camera, which is arranged perpendicular to the light direction (*i.e.*, in the center of the rail). For *θ_i_* < 90°, the white-target should still be illuminated by the lamp, whereas for *θ_i_* ≥ 90° this should no longer be the case. The position and orientation of the lamp is adjusted manually until this criterion is met. The procedure for adjusting *ϕ_i_* is similar.

#### 5.1.4. Sampleholder

The material sample that is presented to the camera is held tightly in place by a separate bespoke sampleholder that is grasped by the robot. Thus, the material can be prepared without a hustle prior to acquisition. The sampleholder has to meet multiple requirements: First, the sample has to be held tight enough, not too move or change shape even in a headlong position. Second, the maximum size is restricted by the robot's working envelope but has to be large enough to contain the spatial variations of the captured material. Thirdly, it should facilitate automatic registration and postprocessing of all captured images.

**Figure 7.**
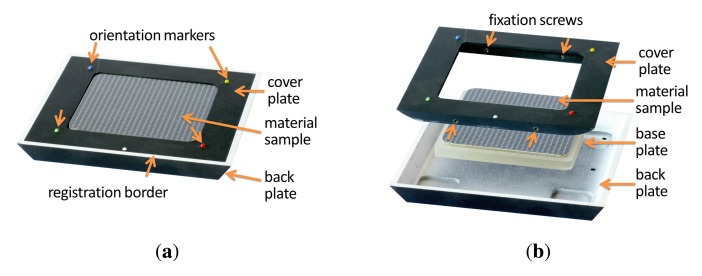
The sampleholder employed in the gonioreflectometer setup. (**a**) sampleholder with sample; (**b**) individual parts.

For this, our sampleholder consists of three distinct parts, a back plate, a base plate and a cover plate, depicted in Figure 7b. The cover plate and back plate are made from aluminium that was milled by a CNC mill. The black coating is achieved by airbrushing the parts with matte black blackboard paint. In contrast to other black spray paint we found the blackboard paint to show virtually no problematic direction depending highlights. A rectangular patch of the material is applied to the base plate made out of acrylic glass. The base plate is embedded into the cover plate, so that the surface of the material is on the same level as the cover. This is then fixated by four screws that penetrate the acrylic glass. Depending on the material, the sample is either held in place by mechanical pressure from the cover plate or it is glued onto the base plate.

The cover plate exhibits several markers aimed to facilitate automatic registration (see Figure 7a). First, the white registration border at the outside that is used to rey the captured images (see Section 4). Furthermore, five differently colored orientation markers are used to verify the orientation and reication.

Over time, several changes have been made to this design (see for example Figure 5). Most notably, the width of the cover plate and registration border has increased to avoid recognition problems during the automatic registration. The sides of the back plate were chamfered to avoid misdetection due to low contrasts under some light directions. Furthermore, the registration border, which originally were white colored stripes on the black cover plate, was separated to the back plate to show a more distinct edge. Eventually, for the spectral measurements in [48], an additional inset with registration borders was added.

From 2004 onwards, all constructed sampleholders have a size of 13 cm × 13 cm and a height of approximately 1 cm to 1.5 cm. In all cases, the cover plate gives room for a 8 cm × 8 cm region of the material sample. With the additional inset (see Figure 5c), the effective sample size is reduced to 6.5 cm × 6.5 cm.

### 5.2. Calibration

Due to the bad repeatability, a full a priori calibration of the cameras is not feasible. However, we calibrate correction factors for the lens distortion by capturing a checkerboard pattern. We do not attempt to recover any other camera parameters or light source positions for single measurements. Since a telephoto lens with a long focal length of 180 mm is employed, we instead assume the camera to be orthographic. Similarly, we consider the light source to be perfectly directional. Note that this is merely a crude approximation. In later setups, described in Sections 6 and 7, we employ the more sophisticated models of finite projective cameras with lens distortions (see e.g., [79]) and light sources with spot light characteristics. Still, for the given distances of 170 cm to the camera and 240 cm to the light source, the direction deviation across the sample is at most 1.9° and 1.4° respectively. This deviation is in the same order of magnitude of the error introduced by the robot arm and rail system (see row “geometric repeatability” in Table 2). Putting more effort into a different camera model would therefore not really improve the precision.

We therefore directly use the given directions of the measurement program as the calibration of the angular domain. However, in order to bring the spatial positions of different images into subpixel precise alignment, we additionally rely on the border-markers found on the target. This registration step is performed as part of the postprocessing after the measurement.

To facilitate the measurement of reflectance values, a radiometric calibration of the setup is performed. First *dark frames* have to be subtracted from all images to correct for hot pixels and sensor bias. Thus, an image *D*_λ_ of the completely unlit room is captured for every wavelength band λ ∈ Λ, using the same exposure time as the BTF measurement. Moreover, the response function *χ* of the camera needs to be inverted to obtain energy values from the pixel values of the raw images. For this, the inverse response function $\chi_{\lambda }^{-1}$ is computed for every wavelength band from shots of a white-standard with varying exposure times [80].

This way, radiance values can be derived from a hyper-spectral image *I* up to a unknown but constant factor *α*_x_**_,λ_**:

(5) $$\alpha_{\text{x},\lambda }L_{\text{x},\lambda }=\frac{\chi_{\lambda }^{-1}(I_{\text{x},\lambda }-D_{\text{x},\lambda })}{T}$$

Here, x denotes an image pixel, *T* the exposure time of the shot and *L*_x,λ_ is the radiance in Wm^−2^sr^−1^ observed in image *I* at pixel x from the current viewing direction *ω_o_*.

Note that *α*_x,λ_ is dependent on wavelength and spatial position in the image. The factor accounts for the mixture of the irradiance of the light source (including attenuation and vignetting), the (spatially varying) opacity of the different spectral filters and vignetting by the camera lens.

To correct for all of these effects at the same time, we capture a set of *white-images W*_λ_ of a white-standard instead of a material sample, using the same wavelengths and direction combinations as the actual measurement. We employ SphereOptics Zenith UltrawWhite^©^ [81], P/N SG3110, which exhibits an almost perfectly Lambertian reflection with about 99% albedo across the visible spectrum. Using *α*_λ_ to denote the known albedo of the white-standard for wavelength λ, we can therefore approximate the reflectance with the constant factor $\frac{\alpha_{\lambda }}{\pi }$. Thus, an irradiance up to a factor of *α*_x,λ_ can be determined by:

(6) $$\alpha_{\text{x},\lambda }E_{\text{x},\lambda }=\frac{\pi }{\alpha_{\lambda }}\frac{\chi_{\lambda }^{-1}(W_{\text{x},\lambda }-D_{\text{x},\lambda })}{T}$$

with *E*_x,λ_ giving the irradiance in Wm^−2^ at the point projected into pixel x, considering a light direction of *ω_i_*

Finally, the spatially varying reflectance samples *ρ*, given in sr^−1^, can be computed as:

(7) $$\rho_{\text{x},\lambda }=\frac{L_{\text{x},\lambda }}{E_{\text{x},\lambda }}=\frac{\chi_{\lambda }^{-1}(I_{\text{x},\lambda }-D_{\text{x},\lambda })a_{\lambda }}{\chi_{\lambda }^{-1}(W_{\text{x},\lambda }-D_{\text{x},\lambda })\pi }$$

Note that for computing the reflectance from image *I*, captured with the angular combination *ω_i_* and *ω_o_*, the corresponding white-image *W* for these directions has to be used. Then the factor *α*_x,λ_ is simply canceled out. More details on this consideration can be found in [71], Appendix 2.

Since the correction with a full set of white-images requires an enormous amount of calibration data and the poor repeatability of the setup complicates a precise spatial alignment, a simplification is proposed in [48]. Instead of all angular combinations, the white-target is only captured under the perpendicular view- and light direction and a single average value over a region of interest in the resulting image is used. The correction with this reduced set of factors neglects any spatial variation in *α* but still yields reasonable results. Nonetheless, the dependency on wavelength is still accounted for.

### 5.3. Measurement Process

The sampleholder with the prepared sample is mounted on the robot. Before beginning the automated data acquisition, the desired ISO speed, aperture and a fixed exposure time per wavelength are chosen manually. Although exposure bracketing could be employed, this has never been implemented. Still, different exposure times are used for the different wavelength bands. All other camera settings remain fixed throughout the measurement. Then a program is started that controls the robot, rail-system, tunable spectral filter and camera. The measurement process is controlled using a single personal-computer. Currently, this is an Intel Core 2 Quad with 2.67 GHz and 2 GB RAM.

The measurement of different angular configurations is completely sequential. Thus, the time necessary for measurement increases linear with the number of angular combinations and quadratic with the number of samples per hemisphere. Hence, we limit ourselves to an angular sampling of 81 directions, *i.e.*, 6,561 combinations. The angular samples are distributed in 6 rings at varying inclination angles *θ*. Each ring is divided into a different amount of azimuthal angles with distance Δ*ϕ* to achieve an even distribution of samples across the hemisphere. Depending on the direction set (see Table 1), the average minimal distance between two sampled directions on the hemisphere is either 14.7° ± 0.4° or 16° ± 0.8° respectively. The directions are not distributed completely uniformly, but the low standard deviations (*i.e.*, ±0.8° and ±0.4°) indicate a good approximation. The selected azimuthal distribution of the samples ensures that for a planar probe the ideal reflection direction is captured. For the 81 configurations with identical light- and view directions *ω_i_* = *ω_o_*, an offset of 10° was added to the light direction, so that the camera would not occlude the light source.

For reaching the different angular combinations the robot arm and camera need to be re-positioned. Moving the robot arm takes between 1 and 5 s. Moving the camera on the rail takes longer, also because the mechanical movements induce vibrations, requiring a waiting period before taking a picture. To minimize delays, the sample points are ordered in that way that in most cases only the wrist of the robot arm needs to be turned to achieve a new azimuth angle. Moreover, the ordering minimizes the movement of the camera on the rail, because this is the most time-consuming operation.

When capturing RGB data, the camera takes a single picture with the predefined exposure time for each angular configuration. The raw images of the Kodak DCS Pro 14n are about 13 MB large, adding up to 83 GB per measurement. Thus, the images need to be directly downloaded to the control PC and stored on the hard disk. Typical measurement times are about 14 h. For a hyper-spectral measurement, it is additionally necessary to tune the spectral-filter to the different bands. The camera is triggered after each filter change. In order not to waste any time, changing of filters runs in parallel to the data transmission. Still, hyper-spectral measurements with 32 narrow bands take 60 h. The images of the Photometric CoolSNAP K4 are 6 MB in size, thus requiring a total of 1.2 TB per spectral measurement.

## 6. Dome 1

**Figure 8.**
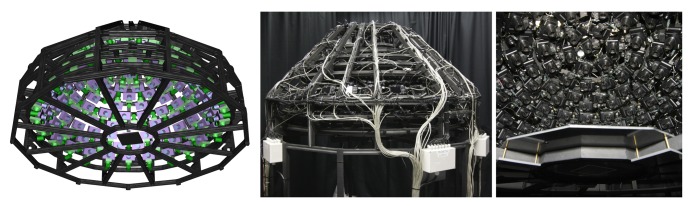
The Dome 1 setup as a schematic illustration (**left**) and in photographs from the outside (**center**) and inside (**right**).

The Dome 1 setup (see Figure 8), constructed in 2004 and published in [5,22,23,72], is a completely view-parallelized BTF acquisition device. To the best of our knowledge, it is the only camera array setup that provides a dense angular sampling without relying on moving cameras or moving the sample. It mounts 151 compact cameras. Between 2008 and 2009 it was completely re-equipped with a new set of cameras. In 2011 [23], it was furthermore extended to support an automated, integrated 3D geometry acquisition based on structured light [83].

**Figure 9.**
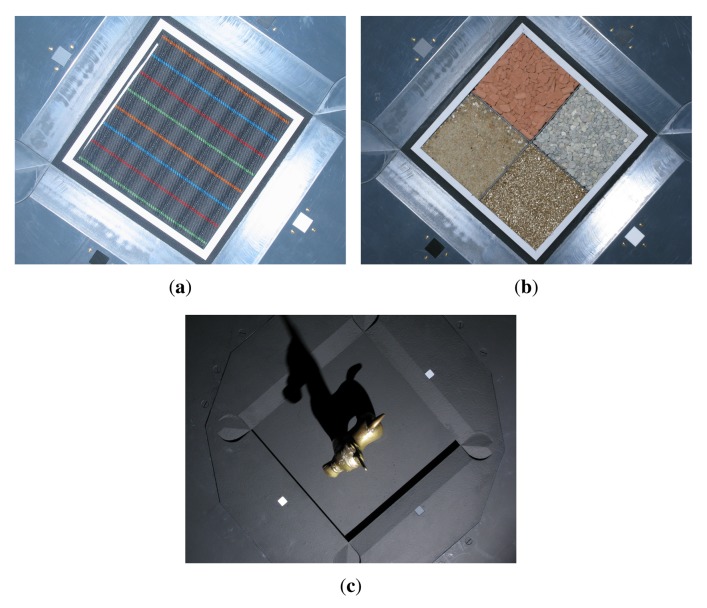
Pictures taken from the topmost camera of the Dome 1. (a) depicts a flat material sample taken with the Canon PowerShot A75 camera. (b) shows four granule material samples captured simultaneously and (c) a 3D object, both captured with the Canon PowerShot G9 camera. The material in (a) is used with courtesy of Volkswagen AG. (**a**) material, PowerShot A75; (**b**) material, PowerShot G9; (**c**) object, PowerShot G9.

Figure 9b demonstrates the capability to capture delicate materials samples, in this case granules and sands, due to the horizontal alignment and rigidity of the sampleholder. Figure 9c shows the acquisition of a complex 3D object.

### 6.1. Hardware

Experience with our gonioreflectometer setup made it very clear that the long measurement time is a major hurdle for BTF measurements that needs to be overcome. Thus, the design goal was a maximal parallelization of the acquisition and complete avoidance of any mechanical movement. At the time of construction, this was already approached by Han and Perlin [18], using a kaleidoscopic setup. Yet, spatial resolution and possible sample sizes were dissatisfactory. In order to allow for practice-oriented sample sizes and resolution in the spatial domain, a hemisphere of cameras was implemented instead. The parallel acquisition with a large number of cameras also holds the advantage that the workload during a measurement equally distributed over many components. This is favorable in terms of durability.

Since a setup without moving parts necessarily only allows for a single, fixed angular sampling, the number of directions on the hemisphere was provisionally increased to 151 rather than the 81 employed in the gonioreflectometer measurements.

#### 6.1.1. Gantry

The 151 cameras are held by a hemispherical gantry structure with an outer diameter of approximately 190 cm, holding the cameras at a distance of 65 cm to the sample. It is organized in 10 camera-rings with different inclination angles from 0° to 75°. Each ring holds a different amount of cameras, distributed across the azimuthal angles with distance Δ*ϕ*. The resulting sampling, shown in Table 3, covers the hemisphere with an almost uniform distribution of directions, having an average minimal distance of 9.4° ± 1°. In azimuthal direction, the Dome's rings are split into 12 segments. The rings are held by 18 vertical struts: one per segment and six additional struts in between each pair. The sampleholder-mount is held by rods, protruding from each of the segments at an inclination of 90° and meeting in the center. The hemispherical gantry with the cameras is standing on 12 legs which are strutted as well for additional stability. In contrast to the original design depicted in Figure 8 (top), two pairs of rods that hold the sampleholder have been removed to allow an operator access to the inside of the dome. To enter, e.g., for placing a material sample or performing maintenance, the operator has to step in through the openings from below. Due to the legs, it is possible to stand upright while working inside.

The frame is made completely from Bosch Rexroth Profiles out of aluminium. It has sufficient strength for holding all cameras and auxiliary components, such as cables, power-couplings or projectors. Due to the many struts, the gantry is perfectly rigid.

#### 6.1.2. Cameras

To keep costs and proportions manageable, we decided to employ compact *point-and-shoot* (P&S) cameras instead of bulky DSLRs. This has the additional advantage that the built-in flashes found in these cameras can serve as light sources. In its first configuration from 2004 [5], the Dome 1 setup was equipped with Canon PowerShot A75 cameras. The CCD sensor has a resolution of 3.2 Megapixel with 10BPP and a Bayer pattern CFA for RGB color. Canon provides an SDK to remotely control the camera via USB, which allows to change focal length, ISO speed, aperture, exposure time and flash intensity (minimum, medium, maximum). It also allows to perform an auto-focus and switching flash exposure on and off.

Setting a focal length of 16.22 mm (35 mm equivalent focal length: 116 mm) for the built-in lens allows to capture a material sample with 235 DPI (see Figure 9a).

However, after about a hundred measurements, the CCD chip of the low-end Canon PowerShot A75 cameras started to fail. Shifted colors, overexposed image regions and clearly visible horizontal stripe patterns appeared. Eventually, the cameras did not produce image content at all. This turned out to be a systematic defect of the camera model [82], being caused by loosening internal wiring of the CCD chip's electronics. Thus, between 2008 and 2009 the Dome 1 was re-equipped with the medium segment Canon PowerShot G9. The latter have a higher sensor resolution of 12 Megapixel with 12 BPP. Although this camera supports to store raw images on the internal memory card, the SDK foresees no way of raw image transmission. We again obtain color-processed and JPEG compressed 8 BPP images. Thus, all radiometric correction steps described in Section 6.2.2. apply for both camera types.

With the PowerShot G9, we capture material samples at a spatial resolution of 450DPI (see Figure 9b) using a focal length of 22 mm (35 mm equivalent focal length: 100 mm). For capturing appearance of complete objects, we adjust the focal length to cover the necessary working volume. Figure 9c depicts an object captured with a focal length of 11 mm (35 mm equivalent focal length: 50 mm), yielding a maximum spatial resolution of 225 DPI.

As the Canon SDK does not give access to the raw sensor data but instead transmits a color-processed and JPEG-compressed 8 BPP image, the camera's response function is not linear. To provide pleasing results close to human perception, the resolution is higher for low energies. When considering the most favorable resolution, the Canon PowerShot A75 captures incident radiance with a dynamic range of 28 dB (at ISO 50) to 21 dB (at ISO 400), but exhibits gross quantization errors of up to 2.4% for the highlights. The situation is almost the same for the Canon PowerShot G9, showing 26 dB (at ISO 80) to 24dB (at ISO 400) with quantization errors of up to 1%. We thus use exposure bracketing with sufficient overlap for capturing high dynamic range values with almost equal resolution. We employ the built-in flash as a light source, which emits a single, strong pulse of light in a fraction of a second. Hence, it is not possible to control the overall exposure using different exposure times. However, we also have to use a fixed narrow aperture of *f*/8 in order to have a sufficiently high depth-of-field. Thus, we instead vary the flash intensity and the ISO speed of the sensors to obtain a multi-exposure image series, yielding a dynamic range of about 33 dB for the PowerShot A75 and 44 dB for the PowerShot G9.

**Figure 10.**
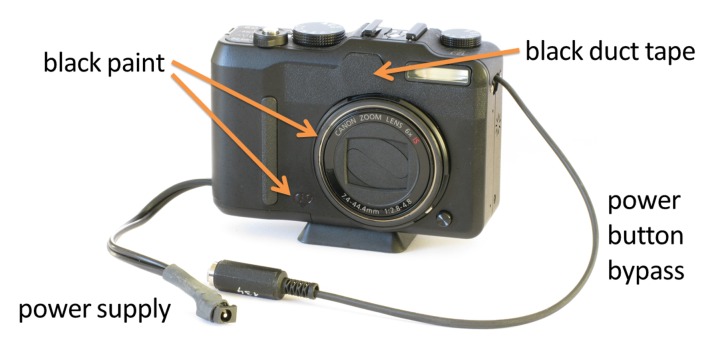
Modified Canon PowerShot G9 camera.

We slightly modified the hardware of the cameras in a few aspects. First, as mentioned in Section 4, we painted reflective surfaces on the front of the camera black and blinded the cameras' auto-focus LED lights with black duct tape. The latter measure also prevents the cameras to confuse each other during the focus procedure. Second, the PowerShot G9 does not provide a jack to support an auxiliary power-supply. At first we employed self-made battery-dummies but eventually found the mechanical contacts to be too unreliable and soldered the power cable directly to the cameras. Finally, we make the power-button of the cameras remotely operable by soldering an additional cable to the button as well. The resulting modifications on a PowerShot G9 camera are shown in Figure 10.

#### 6.1.3. Light Sources

We use the built-in flash lamps of the cameras as light sources. This has several advantages: First, it saves space, wiring and controller logic. Secondly, the camera manufacturer took care that the flash illumination has a well-chosen spectrum to produce natural colors in the images. Thirdly, the flashes have sufficient power for short exposure times, even for materials with low albedo. Finally, in contrast to a strong continuous light source, multiple but very short pulses do expose the material sample to exactly the amount of light necessary for the imaging process, avoiding prolonged UV exposure. Using 151 × 3 flash strobes emits roughly as much UV light (287–400 nm) as a few seconds of an off-the-shelf 100 W tungsten halogen lamp [23].

The built-in flashes are affixed on the camera and close to the lens. Hence, the direction sampling of view and light hemisphere is almost identical. However, we still account for the small difference by separately calibrating the point of origin of the flash illumination. We assume the flash illumination to have a quadratic fall-off behavior with respect to distance and a conic distribution with a cosine fall-off. Unfortunately, the flashes show a low repeatability regarding color and intensity. We therefore measure a correction factor for every single flash exposure and account for it during the radiometric correction of the captured images. More details on this can be found in Section 6.2.2.

We additionally employ a continuous light source that is installed directly above the material sample in the tip of the Dome. The off-the-shelf lamp-socket with a tungsten halogen bulb is remotely toggled using a radio plug. The lamp is used as a light source for camera auto-focus and to verify the correct placement of the sample and focal length of the cameras.

#### 6.1.4. Projectors

For acquiring 3D geometry of objects or material samples we perform an integrated structured light acquisition. We use projectors to impose Gray code patterns onto the object and the 151 cameras capture the illuminated object. We then decode the patterns on the object and triangulate their 3D positions. In order to acquire the complete shape without requiring to reposition the object, we use multiple projectors to provide structured light illumination from all sides. Thus, we installed nine LG HS200G projectors (800 × 600 pixels, LED-DLP, 200 lm); six at *θ* ≈ 82.5° inclination with an even spacing of Δ*ϕ* = 60° and three at *θ* ≈ 17° with Δ*ϕ* = 120°. These particular projector models were chosen because they are compact enough to find a place in the tightly arranged gantry structure, they have a sufficiently near projection distance and large depth-of-field, the LED light source does not produce too much heat and they can almost instantly be switched on and off without long warm-up or cool-down times. They are also reasonably priced consumer products and therefore blend nicely with the rest of the Dome's hardware selection philosophy. Although the resolution of the projectors is rather low, this is compensated by a multi-projector based super-resolution approach [83].

We use Gray code to uniquely idey points on an object surface. Here, the number of patterns depends on the resolution of the projectors. To be more robust, we employ vertical as well as horizontal codes, an additional fully lit pattern and a second pass through the sequence with the inverse of the former signal. We therefore project a total of 2(1 + ⌈log_2_800⌉ + ⌈log_2_600⌉) = 42 images. The projector patterns are provided by a PC via HDMI. Since, by principle, there is always only one projector switched on at a time, we can use a single computer and distribute the signal with a cascade of Aten VS184 4× HDMI splitters. We toggle the projectors by simulating the appendant remote control using computer-controlled infrared LEDs.

Unfortunately, using off-the-shelf consumer projectors also has some pitfalls. We observed that after turning on, the projection drifts and takes up to 15 min to stabilize. Additionally, the colors and intensities alternate periodically with a slightly irregular pattern. Often such a behavior comes from the usage of a color-wheel and can be solved (for black-and-white projection) by removing it. However, our chosen projectors use LEDs with different spectra instead of a color-wheel. This makes it necessary to synchronize exposure with projector frequency in order to avoid intensity shifts. Note that the slowest frequency of the projector irregularities might still be faster than the projectors refresh rate of 60 Hz. We measured the irregularities by deflecting the projection onto a screen with a mirror rotating at 60 Hz. We found although the three primary colors seem to cycle at a higher frequency, the elements of the digital micromirror device produce an irregular pattern that repeats after exactly $\frac{1}{60}s$. Thus, we achieve synchronization by choosing exposure times in multiples of this fraction. This way we ensure that the cameras always integrates over at least one full irregularity period, effectively avoiding flickering.

#### 6.1.5. Sampleholder

**Figure 11.**
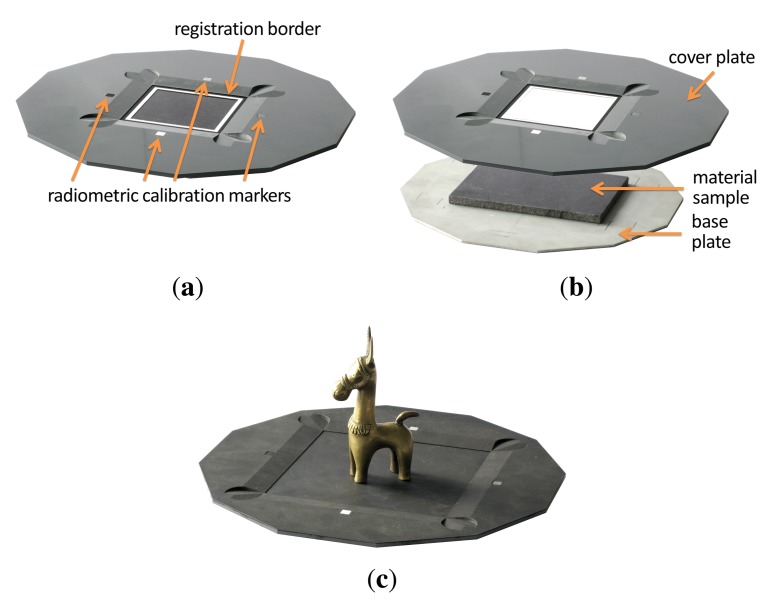
The sampleholder design employed in the Dome 1 setup. (**a**) for materials; (**b**) individual parts; (**c**) for objects.

Since the Dome setup does not require the material sample to be moved, there is no risk that the sample will change shape or get out of place. Thus, it is not necessary to completely mechanically restrain the sample in a sampleholder, as it has been done for the gonioreflectometer. However, for planar material samples we still employ a sampleholder design that combines a base plate with a cover plate, depicted in Figure 11a,b. To prevent curling or wrinkling (e.g., in fabrics or wallpaper), the sample is either fixated on the base plate using double-sided tape or held in place by the weight of the cover plate. The back plate is made from aluminium while the cover plate is made of a hard PVC material. Both are milled with a CNC mill.

The cover plate also contains several markers to facilitate the automatic registration and radiometric correction of the flash illumination. A black-and-white registration border is framing the visible 10.5 × 10.5 cm region of the sample. Four radiometric calibration markers are distributed around the sample. The markers are made from SphereOptics Zenith UltraWhite^©^ [81], showing almost perfectly Lambertian reflectance. We employ a set with four different albedos, P/Ns SG3053, SG3059, SG3080 and SG3102, diffusely reflecting 2%, 10%, 30% and 99% of the visible light.

For the acquisition of 3D objects we utilize a variation of the sampleholder design, which is demonstrated in Figure 11c. The sampleholder is blackened in order not to cast any caustics or indirect light onto the object. Similar to the sampleholder of the gonioreflectometer, we employ airbrushed blackboard paint. There is also no registration border, since the spatial domain of the BTF will be parameterized over the object surface and not a quadrilateral. Yet, the sampleholder has the four radiometric calibration markers, too, since they are necessary to calibrate the flash illumination. The inner frame has an extent of 20.5 cm × 20.5 cm, to allow for a larger acquisition volume in the case of objects.

### 6.2. Calibration

Since the Dome 1 setup is by construction completely rigid and does not require movement of cameras, light sources or sample, we aim to have a more precise calibration than in the gonioreflectometer setup. The position of the cameras, and thus also of the light sources, can be determined a-priori and remain fixed for multiple measurements. The same applies for the radiometric attributes of the CCDs. Unfortunately, the deviations of the cameras' flashes requires a radiometric correction for each exposure. Furthermore, the poor repeatability of the cameras' zoom-lenses and auto-focus as well as some mechanical play in the sampleholder design require an additional fine calibration. This, as well as a registration for a precise alignment, is obtained using white-border markers similar to the ones used for the gonioreflectometer. For 3D objects, a self-calibration of the camera parameters is performed instead, using the structured light features. We dismiss the calibration of the projectors entirely, because of the mentioned problems with the initial shift of the projection.

Eventually, the precise geometric calibration, described in detail in the following section, allows us to employ the models of a perspective camera and a spotlight for determining accurate sample directions for every spatial position.

#### 6.2.1. Geometric Calibration

The procedure to establish an a-priori camera calibration consists of an initial coarse calibration of the extrinsic parameters, which is followed by a subsequent non-linear estimation of the intrinsic parameters. Further, for any given measurement, an additional fine calibration step is performed.

**Figure 12.**
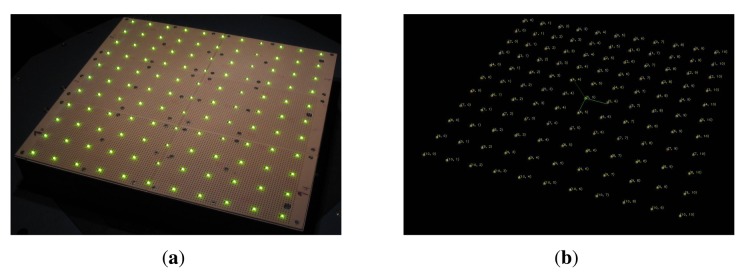
Geometric calibration for the Dome 1 device: (a) shows the 11 × 11 LED calibration target under room light (for illustration purposes). (b) shows a picture taken under calibration conditions with annotation of automatically detected features. The LEDs are aligned on a stripboard in a regular grid with a horizontal and vertical distance of 27.94 mm, spanning a total square of 28 cm × 28 cm. We assume the stripboard to be manufactured sufficiently accurate for our purpose. Pictures taken from [72].

For the initial calibration, we use a planar calibration target with 11 × 11 LEDs (see Figure 12). The target is placed in the center of the Dome instead of the sampleholder. The emitters of the LEDs can be accurately detected in each camera image. We apply prior knowledge about the ideal direction *ω_o_* to resolve the symmetry of the target. The advantage of using LEDs in comparison to typical checkerboard targets is that the emitters can even be robustly detected under grazing viewing angles. Using a fixed planar calibration target, however, is not sufficient for estimating both extrinsic and intrinsic parameters of the cameras. As a consequence, the intrinsic parameters of the 151 cameras are assumed to be identical, which is motivated by the fact that all the cameras are of the same product model. Under this assumption, the calibration method proposed by Zhang [84] can be used to estimate the extrinsic parameters of the individual cameras and the common intrinsic parameters. In the subsequent step, the extrinsic parameters are assumed to be fixed and an optimization of the individual intrinsic camera parameters is performed. For this, the calibration target is captured with the different focal length settings of the Canon SDK. The optimization is initialized with a linear extrapolation of the detected LED-emitter positions using the idealized focal length.

While this calibration procedure yields good and stable results, the intrinsic parameters of the cameras are unfortunately not constant throughout multiple measurements. Although the SDK offers to set the camera to a given focal length, the repetition accuracy of the mechanical zoom for the built-in lens is not precise enough. Furthermore, it is necessary to perform an auto-focus at the beginning of each measurement, also with low repeatability. In practice, the field-of-view differs by a significant amount of pixels. Therefore, a subsequent fine calibration of the camera parameters is performed for every single measurement. This step is performed as post-processing after the measurement, but we will still discuss it as part of the geometric calibration.

In the case of flat material samples, we use the border-markers found on the cover plate (see Figure 11a) for registration and calibration. In contrast to the gonioreflectometer, the sub-pixel precise detection of the material sample region has to be performed only once per view direction instead for every image. As in all our setups, we employ the detected quadrilaterals to rey the spatial samples. Furthermore, we use the corners as a set of accurate and reliable correspondences between the cameras and perform a non-linear optimization [85] to find their respective 3D positions and refine the camera parameters.

When capturing geometry and reflectance of objects, we do not employ border-markers (see Figure 11c). Instead we make use of the structured light patterns for simultaneously reconstructing the 3D geometry and performing a self-calibration of the setup [83]. By decoding the structured light patterns in the 151 cameras, we obtain a large set of reliable and sufficiently accurate correspondences between the views. Given a set of correspondences, it is possible to obtain the depicted 3D geometry and the camera calibration simultaneously using *Sparse Bundle Adjustment* (SBA) [86]. SBA performs a global non-linear optimization, minimizing the re-projection error of the 3D points to the decoded labels in the camera images. However, it requires a good initialization and is susceptible to outliers (*i.e.*, false correspondences due to decoding errors). We therefore follow an iterative approach, alternating between two steps: Fist, we triangulate the correspondences to obtain a 3D point cloud using the given camera calibration. Then, we update the camera calibration and the point cloud via SBA. In the first step, we employ a *Random Sample Consensus* (RANSAC) [87] approach to eliminate outliers. A random subset of 3 cameras is used to triangulate a point and the other correspondences are used to accept or reject the obtained 3D point based on its re-projection error.

As the utilized flash light sources are affixed to the cameras, their positions are given by a fixed offset to the lens. We determined the offset using a ruler. After calibrating the cameras, we apply this offset to the computed center of projection to obtain the light's position. Furthermore, we assume that the light-cone of the flash has the same direction as the cameras' optical axis.

#### 6.2.2. Radiometric Calibration

The radiometric calibration of the Dome 1 device is rather complicated. This is because of the sheer number of employed CCDs, the cameras' inability to transmit their raw data and most importantly the utilization of flash illumination. The cameras' flashes do not show a constant behavior. Instead, color and intensity vary for every discharge. Furthermore, we are forced to use different ISO speed settings and flash intensities for obtaining a multi-exposure series. Each ISO speed again implies a different response function of the CCD. In total, the radiometric calibration of each image depends on the tuple describing a single flash discharge event r = (*f, q, i*), with *f* denoting the flashing camera, *q* the flash intensity quantity and *i* the ISO speed, as well as the response function *χ_c_*_,_*_i_*_,λ_ of the camera *c* taking the picture for color-channel λ. Please refer to Table 4 for a comprehensive overview of all terms and symbols used for describing the radiometric calibration.

We employ a two stage approach for radiometric calibration: In a nonrecurring first step, we calibrate the response functions *χ_c_*_,_*_i_*_,λ_ for each camera *c*, ISO speed *i* and color-channel λ from shots of a white-standard, lit with a continuous illumination, with varying exposure times [80]. Similar to the considerations for the gonioreflectometer, the radiance can be computed from a given image *I^c^*^,r^ that was made with camera *c* at radiometric attributes r = (*f, q, i*) as:

(8) $$\alpha_{\lambda }L_{\text{x},\lambda }^{c}=\chi_{c,i,\lambda }^{-1}(I_{\text{x},\lambda }^{c,\text{r}})$$

with $I_{\text{x},\lambda }^{c,r}$ denoting the pixel for spatial position x and color-channel λ. As in Equation (5), the radiance is only known up to a constant factor *α*. We consider the factor to be spatially uniform because we cannot measure its spatial variation in the Dome 1 setup. Note that we do not divide by the exposure time. This is because we do not employ a continuous light source and hence not an integral over a constant radiance over the time. Instead, the flash discharge can be considered a Dirac delta function, rendering the exposure time irrelevant.

The second step of the radiometric calibration requires to establish the irradiance on the material sample. It is performed for every single flash discharge and is therefore part of the post-processing of a measurement. Because all cameras simultaneously capture images for one particular flash discharge, using the image of just one camera is sufficient for radiometrically calibrating the light source. For this, the pixel intensity values of four radiometric calibration markers attached to the sampleholder (see Figure 11a) are recorded in the image of the topmost camera *c* = 1 (see Figure 9 for examples). We employ multiple markers with different albedos *a_k_* (2%, 10%, 30% and 99% reflectivity) to ensure that at least one marker can reliably be used in a given exposure-image, whereas the others might be underexposed or oversaturated.

For a particular recorded flash discharge r = (*f, q, i*), the idealized irradiance at marker *k* can be predicted using:

(9) $${Ê}_{\text{r},k}=\left(\frac{{\text{v}}_{f,k}}{{\left\|{\text{v}}_{f,k}\right\|}_{2}}\cdot {\text{n}}_{k}\right)\;{\left(\frac{d}{{\left\|{\text{v}}_{f,k}\right\|}_{2}}\right)}^{2}\left(\frac{{\text{v}}_{f,k}}{{\left\|{\text{v}}_{f,k}\right\|}_{2}}\cdot {\text{n}}_{f}\right)$$

Here, the first dot product models the foreshortening of the light direction according to Lambert's cosine law. The second term models the quadratic light fall-off. The last term models the fall-off due to the conic shape of the flash. In our implementation, the quadratic light fall-off is normalized to a distance of *d* = 65 cm, which approximately corresponds to the inner radius of the Dome 1 device.

A correction factor *β*_r,λ_ describing the variance of a particular flash discharge can be obtained by taking the weighted average over all four markers:

(10) $$\beta_{\text{r},\lambda }=\frac{1}{\sum_{k}w({\bar{m}}_{\text{r},k})}\sum_{k}w({\bar{m}}_{\text{r},k})\frac{a_{k}}{\pi }\frac{{Ê}_{\text{r},k}}{\chi_{1,i,\lambda }^{-1}({\bar{m}}_{\text{r},k})}$$

where *m̄*_r,*k*_ denotes the average pixel value for marker *k*. The term $\frac{\alpha }{\pi }$ models the Lambertian reflectance of the respective marker. *w* is a weighting function to omit over- and underexposed markers from the computation of the factor. In the event of capturing 3D objects, one of the markers can be in shadow for some light directions (see Figure 9c). This needs to be accounted for by applying a weight of zero in these cases.

Using the correction factor, the true irradiance at spatial position x can be described as:

(11) $$\alpha_{\lambda }E_{\text{r},\text{x}}:=\beta_{\text{r},\lambda }{Ê}_{\text{r},\text{x}}$$

The constant factor *α*_λ_ is contained because *β* is normalized by $\chi_{1,i,\lambda }^{-1}({\bar{m}}_{\text{r},k})=:\alpha_{\lambda }L_{k,\lambda }^{1}$, *i.e.*, the radiance from marker *k* up to this factor.

Finally, the high dynamic range reflectance values for a given combination of capturing camera *c* and flashing camera *f* can be obtained by combining the multiple differently exposed pictures *I^c^*^,r^ in a weighted sum (similar to [80]):

(12) $$\rho_{\text{x},\lambda }=\frac{1}{{\text{∑}}_{q,i}w\left(I_{\text{x},\lambda }^{c,\text{r}}\right)}\underset{q,i}{\text{∑}}w\left(I_{\text{x},\lambda }^{c,\text{r}}\right)\frac{\chi_{c,i,\lambda }^{-1}\left(I_{\text{x},\lambda }^{c,\text{r}}\right)}{\beta_{\text{r},\lambda }{Ê}_{\text{r},\text{x}}}=\frac{1}{{\text{∑}}_{q,i}w}\underset{q,i}{\text{∑}}w\frac{\alpha_{\lambda }L_{\text{x},\lambda }^{c}}{\alpha_{\lambda }E_{\text{r},\text{x}}}$$

As can be seen, the factor *α*_λ_ is canceled out.

**Figure 13.**
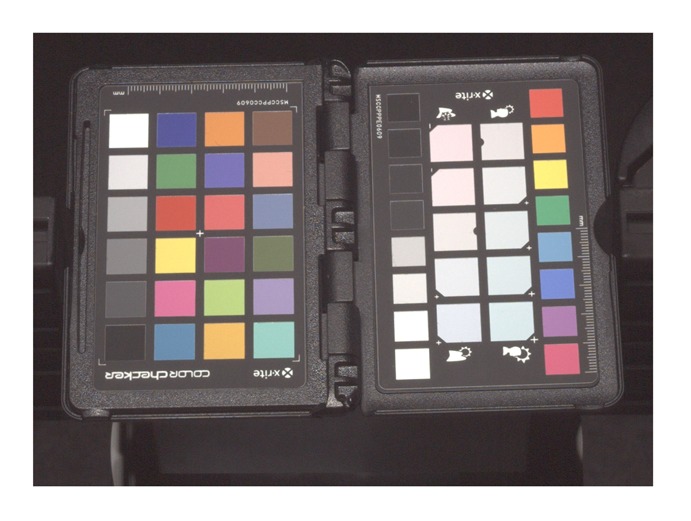
The X-Rite ColorChecker Passport. The color chart is used to calibrate the cameras' color profiles in the Dome 1 and Dome 2 as well as the cameras' response functions of the Dome 2.

Although the camera images' EXIF data specifies that the colors are given in sRGB, a comparison with hyper-spectral measurement data indicates that Canon applies additional color processing, such as intensifying the saturation for some colors. We therefore perform an additional color calibration. We use a X-Rite ColorChecker Passport color rendition chart (see Figure 13) to establish the CIEXYZ color profile for each camera.

### 6.3. Measurement Process

Despite the construction with twelve segments, the Dome is logically divided into eight azimuthal parts. Each octet consists of 19 or 18 cameras and has a separate power supply and control PC. The current control PCs are each equipped with an Intel Core 2 Quad CPU with 2.33 GHz, 1.75 GB RAM, a NVIDIA GeForce 9300 GPU and a 1 TB hard-drive. The camera and flash settings are controlled via USB and the captured images are directly transmitted to the respective computer.

Since the setup is rigid, the homography for spatial registration can be computed as soon as the focal length and auto-focus are set. Thus it is possible to directly perform the reication during measurement in case of flat materials. The control PCs have a sufficient computational capacity to process the 19 incoming images on the fly using CUDA on the GPU. Nonetheless, the raw measurement data is stored on disk as well. The first of the client computers is furthermore used to show the patterns for structured light via HDMI.

The overall acquisition process is controlled via a ninth host computer that is connected to the clients via 100 MBit/s Ethernet. The master computer is also responsible for the continuous auto-focus light source, the remote control of the projectors and switching the camera power on and off.

After all cameras have been turned on and detected by their respective control computers, some basic camera settings, such as white-balance, shutter-speed and aperture, are applied. The focal length is adjusted for the measurement task: 16.22 mm for the PowerShot A75 or 22 mm for the PowerShot G9 for materials samples, a flexible focal length for objects. Then, the auto-focus procedure is performed and locked. For this, we shortly activate the continuous light source. In case the material sample shows a low contrast for some of the cameras, we place a printed black-and-white focus target on the material and remove it after the successful auto-focus.

For capturing the HDR reflectance, a set of LDR sequences with different ISO speeds *i* and flash intensities *q* is shot. We employ the lowest ISO speeds whenever possible, since they provide a better signal to noise ratio. Only if the dynamic range of the material reflectance exceeds the dynamic range of the flash intensities, we switch to higher ISO settings as well. We implement the measurement program for a single LDR step (*i, q*) by first setting the ISO speed *i* and flash intensity *q* for all cameras. All flashes are pre-charged for a fast response, but set not to discharge with the exposure. Then, we loop through each flash *f* ∈ [1,…, 151]. Camera *f* activates the flash. All cameras are triggered to simultaneously take a picture; during this, camera *f* will flash, since it has been activated. Finally, the flash for camera *f* is deactivated and the loop continues with the next camera. Note that this procedure requires only 151 flash discharges per 22,801 images.

Since the cameras are controlled using different computers and via a USB connection, it is not trivial to synchronize the exposure of all other 150 cameras with the one camera that will flash. To tackle this, we use a rather long exposure time of 1 s for the PowerShot A75 and 2.5 s for the PowerShot G9. Camera *f* is triggered 0.5 s after the others, so the flash definitely falls within the exposure interval. The cameras directly transfer the image data to the control PCs in JPEG format. The images of the PowerShot A75 camera have an average filesize of 251.2 KB and can be transmitted in about 10 s. The 12 Megapixel PowerShot G9 requires an average of 3.16 MB per image and the transmission takes about 9 s. Interestingly, in both cases, the total time amounts to about 11 s per light direction. Capturing one full LDR sequence takes 27–28 min. We typically utilize four combinations of ISO speed and flash quantity. Thus, a total of 91,204 raw images are captured in 1.8 h. The raw data sizes are 21.85 GB for the PowerShot A75 and 281.15 GB for the PowerShot G9, respectively.

As described earlier, we optionally perform a structured light acquisition to obtain accurate an 3D geometry of the sample. The details of the 3D reconstruction can be found in [83]. We capture the structured light patterns using exposure bracketing. Since the projectors are continuous light sources, varying exposure times can be applied for this. Therefore, we set the cameras to the lowest ISO speed and take multiple sequences of Gray code patterns *g* ∈ [1, 2,…, 42] from all projectors *p* ∈ [1, 2,…, 9] using different exposure times *t*.

Switching the projectors on and off takes the longest and is thus performed least frequent. First, the current projector *p* is powered on and all cameras are set to exposure time *t*. Then we project each pattern *g* and take pictures of the pattern illuminated object with all cameras simultaneously. After all patterns have been displayed, we proceed with the next exposure time. When all exposure sequences are captured, we power off the current projector and repeat the procedure with the next one.

Here, we do not need to be too careful about the synchronization. Instead, we wait 100 ms after each pattern-change and then directly capture the image with all cameras simultaneously. We proceed with the next pattern as soon as all cameras have finished transmitting their images. The shorter exposure times make the process faster. The transmission of the images is faster as well: Due to the Gray code illumination, more than half of the image content is black, resulting in nearly half of the average file size of 1.7 MB for the JPEG images. The time for the transmission is about 4 s. In most of our structured light measurements, we employ 3 different-exposure times *t* ∈ {50 ms, 125 ms, 500 ms}. This results in a total of 171,234 images, which are captured in about 1.4 h.

Note, however, that the captured data is stored fragmented over 8 control PCs and needs to be copied to its permanent storage destination after the acquisition. We refrain from a transmission during measurement to avoid synchronization issues due to lags in the network communication with the master computer.

## 7. Dome 2

**Figure 14.**
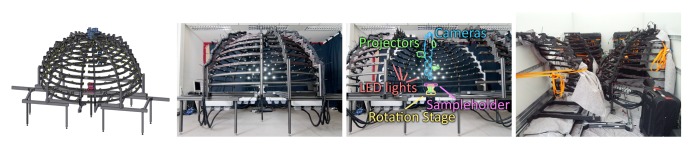
From left to right: The Dome 2 setup as a schematic illustration, photographed in the closed and fully opened configuration as well as disassembled and packed into a light commercial vehicle.

The Dome 2 setup (see Figure 14), published in [71,73,74], is a camera array setup that combines a fixed light dome, comparable to the Dome 1 setup or [57–61,69,70], with a multi-camera arc and a turntable, similar to [21,64,65]. It was built between 2011 and 2012 with the goal to combine the strengths and overcome the shortcomings of the two previous setups. The Dome 2 was designed to facilitate integrated 3D acquisition from the start. Thus, the sample is always leveled, as in the Dome 1 setup. However, similar to the gonioreflectometer, we now employ high-end cameras and well-behaved continuous light sources to avoid the calibration issues of the first Dome.

We also included the experience gained with user-requirements in our design. The Dome 2 setup is capable of reliable non-stop measurement operation. The design foresees the possibility of an automatic feed for material samples and the easy and fast deployment of the setup off-site.

### 7.1. Hardware

After more than five years in use, the weaknesses of the consumer grade point-and-shoot cameras in the Dome 1 device became very apparent. Thus, the new design consequently employs high-end industrial parts. However, this decision would make the construction of another complete camera hemisphere prohibitively costly. We therefore employ a hybrid approach that still features some parallelism in the view direction sampling: We equip a quarter circle above the material sample with 11 cameras, which observe the sample from different inclination angles *θ_o_* in parallel. A turntable is used to achieve a sampling of different azimuthal directions *ϕ_o_*. To keep time-consuming mechanical movement to a minimum, we employ a full hemisphere of 198 rigidly positioned light sources, avoiding any movement when sampling the light directions. As with the Dome 1, most of the angular resolution is thereby again predetermined by the hardware. However, the proposed arrangement shows yet another increase in angular resolution and the turntable provides additional flexibility for balancing azimuthal resolution and measurement speed. Note however, that, due to the rigid arrangement of lights and cameras, the azimuthal sampling of the view direction and of the light direction are not independent. We eventually use 198 × 264 directions (see Table 5), instead of 151 × 151 or 81 × 81.

#### 7.1.1. Gantry

Similar to the Dome 1 setup, all components are held by a hemispherical gantry. The gantry is again made from Bosch Rexroth profiles and organized in rings that are held rigidly by nine vertical struts. However, the Dome 2 has a larger inner diameter of 2 m. Due to this and because the cameras are only arranged on an arc, the rings can now be spaced evenly at the inclination angles *θ* = 0°, 7.5°,…, 90°. The eleven cameras are installed on the rings from *θ* = 0°, 7.5°,…, 75° right below each other. On every second ring the cameras are displaced by *φ_o_* = 7.5° to have enough space. The different azimuthal angles are reached using a turntable in Δ*ϕ_o_* = 15° steps. The resulting view directions, listed in Table 5, are distributed slightly denser than in the Dome 1 setup and have an average minimal distance of 7.6° ± 2.6°. Note that the higher standard deviation indicates a less uniform distribution of the directions.

198 LED lamps are installed on the rings as well. 188 of them are placed in equidistant azimuthal angles Δ*ϕ_i_* to achieve an even sampling over the hemisphere with an average distance of 9° ± 1.2°. They are aligned symmetrically around the camera on the respective ring by applying an azimuthal displacement of $\varphi_{i}=\frac{1}{2}\Delta \phi_{i}+\varphi_{o}$. This arrangement was chosen, because it facilitates the acquisition of reciprocal image pairs for turntable rotations of *n* · Δ*ϕ_i_* + *φ_i_*. In turn, this allows to use the Helmholtz reciprocity principle in 3D reconstruction [73]. Note that the spacing of the light sources with *Δϕ_i_* as multiples of 15° causes the light direction samples to be mostly identical for the different turntable rotations with Δ*ϕ_o_* = 15°. Another 10 lights are placed at the perfect mirror direction of the cameras, *i.e., ϕ_i_* = *ϕ_o_* + 180°. See Table 5 for a detailed listing.

The construction consists of four basic parts: A base, standing on nine legs, on which two quarters and one half of the dome are mounted. For transportation, the frame can be quickly disassembled into these parts and packed into a light commercial vehicle. All of them fit through standard doorframes. When assembled, the quarters of the hemisphere can be slid open, giving access to the inside. Figure 14 shows the Dome 2 setup in all three configurations. There is also enough space to let an automatic feed pass through below the ring at 90° inclination, for continuously measuring several material samples in sequence.

#### 7.1.2. Cameras

We employ SVS Vistek SVCam CF 4022COGE industrial video cameras. The CCD-sensor has a resolution of 4 Megapixel with 14 BPP. It has a quadratic shape of 16 × 16 mm, which reduces the amount of pixels that do not display the material sample in an image. Like the DSLR cameras of the gonioreflectometer, the Vistek cameras have a Bayer-patterned CFA to measure RGB color. The large pixels show a high light sensitivity, *i.e.*, low noise levels, providing a high dynamic range of about 32 dB per image. We additionally use exposure bracketing to account for higher dynamic ranges. For this, the electronic shutter has customizable exposure times from 50 *μ*s to ∞. The cameras are connected via Gigabit-Ethernet and are capable of transmitting up to eight images per second with 12 BPP. All eleven cameras are operated by a single computer, avoiding any synchronization issues or the fragmented storage of the captured data.

The cameras are equipped with high-quality ZEISS Makro Planar T*2 ZF-I prime lenses. Aperture and focus can be fixated using locating screws. Therefore, all lens-dependent intrinsic parameters are constant, vastly improving the stability of camera calibration and its validity throughout multiple measurements. For measuring flat material samples we employ a focal length of 100 mm (35 mm equivalent focal length: 190 mm), offering approximately 380 DPI spatial resolution. For the acquisition of larger 3D objects, we exchange the lenses with a second set of 50 mm focal length (35 mm equivalent focal length: 95 mm), providing 190 DPI. In both cases, we use a fixed aperture of f/19 on all lenses to have a sufficiently large depth-of-field and focus on the center of the Dome 2 setup.

**Figure 15.**
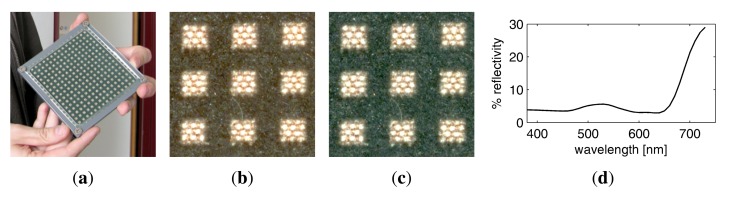
Color reproduction of a green fabric material captured in the Dome 2 setup. (a) shows a picture of the material taken with a P&S camera under natural lighting. (b) and (c) are images taken with a Vistek camera without and with the IR cut-off filter, respectively. Note how in (b) the material appears to have a red tint. The measured reflection spectrum (d) of the green part shows a significant peak in the infrared. (**a**) photograph; (**b**) without IR filter; (**c**) with IR filter; (**d**) reflectance spectrum.

**Figure 16.**
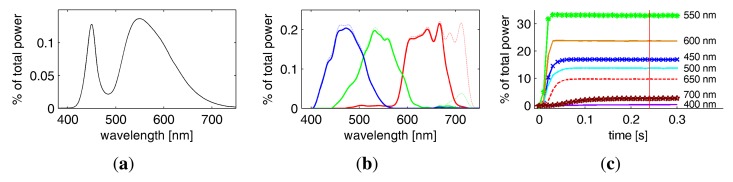
Spectral power distribution of the employed LEDs (a) and sensitivity of the cameras (b). The red, green and blue curves correspond to the respective primaries in the Bayer pattern. Dashed curves indicate the response without the IR cut-off filters. In (c) the change in spectral power distribution of a LED after activation (at 0 s) is shown. Higher wavelengths take longer to reach their final power output. The vertical red line at 240 ms marks the time at which the 99th percentile of the final power is reached. After this point we consider the spectral characteristics to be stable. (**a**) LED; (**b**) Camera; (**c**) LED time series.

In contrast to the consumer photo cameras employed on the gonioreflectometer and Dome 1, the CCD sensor of the Vistek cameras does not have an infrared cut-off filter. However, as illustrated in Figure 15, blocking the near-infrared is important to preserve the natural color impression to a human observer. We therefore use additional B+W 486 UV/IR cut-off filters on our lenses. Figure 16 demonstrates the spectral sensitivity of the employed camera with and without the filter.

#### 7.1.3. Light Sources

To avoid the problems encountered with flash light sources in the Dome 1, such as the complex radiometric calibration and the inconvenient exposure bracketing, as well as to provide the necessary amount of 198 light sources, we employ LED lamps as an inexpensive and reliable solution for continuous illumination. The decision for LED lamps inferred two additional considerations: We wanted to use single emitter LED lamps to be able to presume an ideal point-light illumination for computing the light directions. Further, the LEDs should be phosphor-coated to exhibit a continuous spectrum rather than three narrow peaks, facilitating a natural image impression. We selected Barthelme Bari DC 2.5 W show-case LED lamps (215 lm). Their 2.5 W LED emitter was amongst the most powerful available at the time. In addition, the lamps come with lens-optics to achieve a spotlight characteristic, concentrating most of the emitted radiance on the material sample. We account for the strong spatial variance of the illumination in our radiometric calibration procedure.

Although the LEDs have an uneven spectral distribution, there are no holes in the spectrum (see Figure 16a) and most of the power is actually concentrated in the spectral bands to which the cameras are sensitive. All LEDs are from one batch to avoid differences in brightness and spectra with a color temperature of 6, 000 K. Additionally, after switching on an LED we wait for 250 ms for it to reach stable operating conditions and spectral characteristics (see Figure 16c).

#### 7.1.4. Projectors

Similar to the Dome 1, the Dome 2 setup is equipped with four digital projectors for an integrated 3D reconstruction via structured light [73,83]. The projectors are installed next to the camera arc at different inclination angles between 0° to 90°.

The setup at the University of Bonn is still equipped with LG HS200G LED projectors. However, due to the projectors shortcomings, discussed in Section 6.1.4., we propose to replace them with CASIO XJ-A141 (1, 024 × 768pixels, LED-DLP, 2,500lm) models. In our first experiments, the Casio projectors do not exhibit a drift and also support shorter synchronization times.

#### 7.1.5. Turntable & Sampleholder

**Figure 17.**
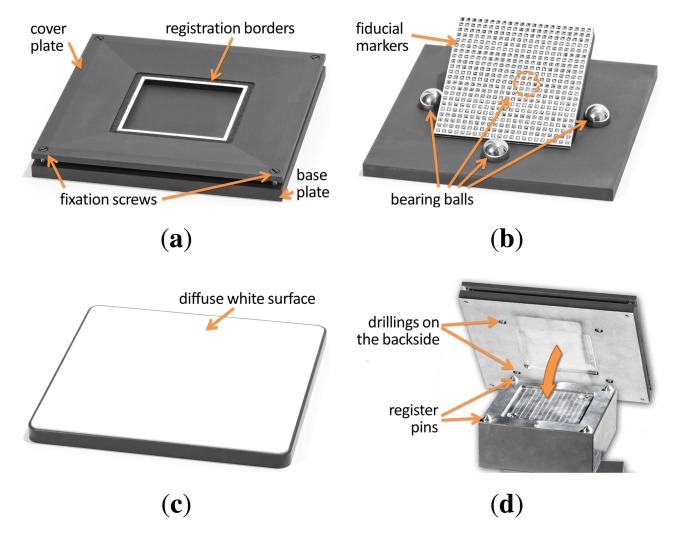
The Dome 2 sampleholder (a) and custom-tailored geometric (b) and radiometric (c) calibration targets. All three are fixated using the mechanism shown in (d). (**a**) sampleholder; (**b**) geometric target; (**c**) radiometric target; (**d**) fixation on turntable.

To achieve the different azimuthal view angles, we utilize a Newport URS-150BCC computer-controlled precision rotation stage with a guaranteed uni-directional repeatability of 0.002°. This is in agreement with the results of our experiment, sketched in Section 8.1. Here, we obtained an average pose deviation of 0.0035° for repeatedly capturing the same sequence of rotations. We limit the maximum rotation speed, acceleration and deceleration to avoid shifting or deforming the sample. Rigidly attached to the turntable we installed a vertical-stage dummy with four conic register pins on top (see Figure 17d). The register pins interlock with drilled holes on the backside of our sampleholders and calibration targets. This way, the sampleholders and targets can be exchanged and put back into exactly the same position. The fixation is rigid and has virtually no mechanical play, ensuring a high precision and repeatability.

Flat material samples are fixated on a blackened sampleholder (see Figure 17a). If necessary, the sample can be glued to the base plate with double sided tape as in the previous setups. A cover plate is put on top of the material sample and provides an adjustable clamping pressure via four screws. All parts of the sampleholder and the fixation mechanism are made from aluminium with a CNC mill. Following our experience from the previous two setups, we again employ airbrushed matte blackboard paint as black coating. In contrast to the cover plate of the Dome 1 setup, the adjustable clamping pressure avoids squeezing of soft materials, which could otherwise change the appearance. The visible area of the material sample is 7.5 cm × 7.5 cm. The black and white registration borders framing the material are used for the automatic registration. For capturing 3D objects, we simply place them on the base plate without applying the cover plate. This is possible because, in contrast to the Dome 1, calibration markers are not required.

### 7.2. Calibration

One huge advantage of the Dome 2 setup is the fact that all components show a high repeatability. First, most of the hardware is rigidly affixed, even the focal length, aperture and focus distance of the camera lenses. Second, the only movable components, *i.e.*, the rotation stage and detachable sampleholders, show a high precision and good repeatability. Radiometrically, the situation is the same with the LED lights reaching a stable and repeatable state very quickly. This allows to perform a single accurate calibration that remains valid as long as the hardware components are not disrupted, eliminating the need for an additional refinement of the calibration per measurement. We currently do not calibrate the projectors, due to the problems with the initial shift of the projection described in Section 6.1.4.

#### 7.2.1. Geometric Calibration

In contrast to the Dome 1, the cameras capture the sample at multiple turntable positions. Hence, a registration of the different rotated acquisitions is required. For this purpose, it is necessary to calibrate the rotation axis and center of the turntable in addition to the parameters for the cameras and light sources. Further complexity of calibration is introduced by the fact that the positions of light sources and cameras are decoupled in this setup. This prevents the solution found for the flashes of the Dome 1 of adding a known offset. Instead, the light sources are calibrated independently.

The geometric calibration of all parts is performed utilizing a custom-tailored target (see Figure 17b) that consists of a plate with fiducial markers [88] and four polished bearing balls. The calibration target is designed to fill most of the cameras' field of view. For the 50 mm lenses we employ a target with an extent of 25cm × 25cm, whereas the target for the 100mm lenses is 18cm ×18cm in size. Markers and balls have a known size and position. We employ bearing balls with a diameter of 50 mm and 20 mm respectively. The target is rotated by the turntable for being captured in various different poses.

We first calibrate the cameras and the turntable using the fiducial markers. The markers are uniquely ideiable and orientable and have a Hamming-distance of 3, avoiding accidental misclassification. By subpixel accurately detecting the corners of the markers, we obtain a large set of highly reliable homologous points (4 per marker) between the different poses of the target as well as the different cameras. Using these correspondences, we first employ Zhang's algorithm [84] to obtain an initial guess for performing a consecutive bundle adjustment [86]. The resulting reprojection errors are 0.16 pixels on average, which corresponds to a spatial error of 11 *μ*m (for the 100 mm lenses) and an angular error of 0.001° in the view direction. The turntable's axis and center of rotation are obtained from the triangulated 3D locations of the markers' corner points. After calibration, different poses can be brought into alignment with an average deviation of 0.003°. This is at the level of the repetition accuracy of the turntable and therefore sufficiently accurate.

For calibrating the light positions, we idey for each light its reflection point in all four bearing balls. Let l*_l_* ∈ ℝ^3^ denote the true position of the LED *l* (in our setup, *l* ∈ 1,…, 198;, then ${\text{l}}_{l,c,b}^{\prime }\in {\text{R}}^{2}$ is the detected position of its reflection in ball *b* in the image taken by camera *c*. We describe the bearing balls via their center c*_b_* and their fixed diameter. Because the marker plate can cause shadowing and occlusion for some combinations of light sources and cameras, we capture the balls under a sufficient number of rotated poses. These can be thought of additional “virtual” bearing balls where the center c*_b_* is described by the rotation ${\text{c}}_{b}=\text{R}(\alpha ){\text{c}}_{b}^{\prime }$ of an unrotated bearing ball *b′* for an angle *α*. Without loss of generality we use *b* = 1,…, 4 to refer to the unrotated bearing balls.

Using these features and a good initial estimate for the bearing balls' positions, we can compute the reflection rays via ray-tracing and triangulate the LED position from them. Afterwards, we perform a non-linear optimization on all LED locations and the unrotated sphere positions simultaneously to reduce the re-projection error of the observed reflections:

(13) $$\underset{{\tilde{\text{c}}}_{1},\ldots ,{\tilde{\text{c}}}_{4},{\tilde{\text{l}}}_{1},\ldots ,{\tilde{\text{l}}}_{198}}{\arg\min}{\underset{l,c,b}{\text{∑}}\left\|{\text{l}}_{l,c,b}^{\prime }-{\tilde{\text{l}}}_{l,c,b}^{\prime }\right\|}^{2}$$

Here, ${\tilde{\text{l}}}_{l,c,b}^{\prime }$ is the projection of the reflection of LED *l* with estimated position l̃*_l_* in bearing ball *b* with estimated center c̃*_b_* into the camera image *c*. Note that we only need to consider the centers of the unrotated bearing balls during optimization as those for other rotations are directly derived from them. We employ the Levenberg-Marquardt algorithm [85] to find a solution. The optimization terminates after about 50 iterations, taking a total of about 20 min on our processing computer (see Section 7.3).

A similar approach was recently published by Ackermann *et al.* [89]. Here, the authors used a single camera and did not include the position of the balls in their optimization. With our global optimization of all parameters, we can report an average error of 0.4pixels, corresponding to an angular error of about 0.08° for the light directions.

#### 7.2.2. Radiometric Calibration

The radiometric calibration of the Dome 2 is closely related to the radiometric calibration performed in our gonioreflectometer setup, described in Section 5.2. The situation for a single direction combination is very similar: The Vistek cameras provide us with a 12 BPP raw data from the CCD and the employed LED light sources are continuous light sources with a constant illumination. Please note that we are currently not considering long-time degradation effects on the light yield, which could be antagonized by periodic radiometric re-calibration.

Following the procedure of the gonioreflectometer setup, we first take *dark frames D_c_*_,_*_T_* for every camera *c* to correct for hot pixels and sensor bias. Although the Vistek cameras capture RGB color, this is achieved using a color filter array in front of the sensor. Thus, the obtained raw images are monochromatic prior to demosaicking, similar to the situation of the hyper-spectral gonioreflectometer. However, while the gonioreflectometer employs different exposure times for each wavelength band, this is not the case in the Dome 2 setup. Hence, in contrast to the procedure described in Section 5.2, we do not need to account for a wavelength band or color-channel λ. Instead, we take a multi-exposure series with different exposure times *T*. We use linear interpolation between the dark frames *D_c_*_,_*_T_* to perform dark-frame subtraction for arbitrarily exposed measurement images. We further compute the response function *χ_c_* for every camera by employing the method of Robertson [80], taking an exposure series of an X-Rite ColorChecker Passport color rendition chart (see Figure 13) that is placed on the sampleholder's base plate. The color chart is chosen as a target because it provides favorable variations in intensity and hue. Similar to the Dome 1 calibration, we also use the color chart to establish the CIEXYZ color profile for each camera.

For a given camera *c* and LED lamp *l*, this allows us to obtain radiance values up to a unknown but constant factor *α*_x,_*_l_*_,_*_c_*. For the sake of better readability, we dismiss the camera and lamp indices in the following equations. Similar to the Dome 1 setup, we use multiple exposures to increase the available dynamic range. For a given series of images {*I^T^*}*_T_* with exposure times *T* ∈ ℝ^+^, we obtain the radiance *L*_x_ of the sample point observed in pixel x into the direction of the camera using the weighted sum (similar to [80]):

(14) $$\alpha_{\text{x}}L_{\text{x}}=\frac{1}{{\text{∑}}_{T}w\left(I_{\text{x}}^{T}\right)}\underset{T}{\text{∑}}w\left(I_{\text{x}}^{T}\right)\frac{\left(I_{\text{x}}^{T}-D_{\text{x}}^{T}\right)}{T}$$

Here, $I_{\text{x}}^{T}$ denotes the value of the pixel x in the captured image and $D_{\text{x}}^{T}$ denotes the value of the same pixel in the linearly interpolated dark frame.

We obtain the radiance for different color-channels λ ∈ {red, green, blue} by demosaicking *L* according to the Bayer pattern of the Vistek's CFA, using the method of Lu and Tan [90].

Similar to the radiometric calibration of the gonioreflectometer, we capture a set of camera and LED dependent *white-images W_c_*_,_*_l_* of a white-standard (see Figure 17c). The white-standard is made of Labsphere Spectralon^©^ [91], P/N SRT-99-100, which is almost perfectly Lambertian with an albedo *a* of 99% in the visible spectrum. With it, the irradiance of the light source can be determined up to the factor of *α*_x_ by:

(15) $$\alpha_{\text{x}}E_{\text{x}}=\frac{\pi }{a}\frac{\chi^{-1}\left(W_{\text{x}}-D_{\text{x}}^{T}\right)}{T}$$

Due to the Lambertian reflectance of the white-standard, using a single exposure time *T* is sufficient to capture its full dynamic range.

When placed on the turntable, the surface of the white-standard is at the same level as a material surface would be during measurement. Given the precise repeatability of the Dome 2, the factor *α*_x_, which implicitly contains spatially varying illumination effects such as vignetting, chromatic aberrations or distance fall-off, is therefore exactly the same in the white-images and the measurement images. By using the corresponding pair of measurement image $I_{c,l}^{T}$ and white-image *W_c_*_,_*_l_*, the factors *α* are canceled out. All spatially varying illumination effects are therefore corrected without the need for an explicit model and the spatially varying reflectance sample *ρ* at pixel x is given as:

(16) $$\rho_{\text{x}}=\frac{L_{\text{x}}}{E_{\text{x}}}=\frac{\alpha_{\text{x}}L_{\text{x}}}{\alpha_{\text{x}}E_{\text{x}}}$$

More details on this consideration can be found in [71], Appendix 2.

We observed that the variation in illumination over the surface is low-frequent. To save memory, we therefore store the white-images *W* reied to the quadratic 3D surface of the white-standard in a low resolution.

Note that the implicit correction is not possible for points that significantly protrude from the surface of the white-target. Plans are in place to capture the white-target at different heights and use a trilinear interpolation for the radiometric correction on 3D objects. However, currently we instead assume a conic shape of the light distribution and hence extrapolate the value of *W*_x_ along the ray to the light source position to obtain a volumetric correction factor. Here, we also account for the quadratic fall-off with respect to distance to the light source.

### 7.3. Measurement Process

The measurement is controlled by a single computer that is equipped with two Intel Xeon E5620 CPUs with 2.4GHz, 24GB RAM, an NVIDIA GeForce GTX 460 GPU and 12 Gigabit Ethernet ports. Similar to the Dome 1, the computer is capable of performing reication, HDR combination and radiometric correction on-the-fly during the measurement. Nonetheless, all raw images are written to disk as well.

The measurement data is captured directly onto a freshly formatted hard-disk with 2 TB or 3 TB to avoid loosing write-speed due to file-system fragmentation. Still, depending on the exposure time the data rate can reach 528 MB/s and the disk's write-speed becomes a limiting factor for the measurement performance. Therefore, we employ a write-queue in RAM, which is worked off during more time-consuming operations. After the measurement, the hard-disk, which is mounted in a hot-swap drive-bay, can be swiftly exchanged to enable further measurements without delay. This improves upon the Dome 1, where the measurement data is fragmented over eight PCs and has to be copied to a permanent storage destination over the network.

For flat material samples, pictures of the border-markers on the cover plate of the sampleholder are taken under all 24 rotations. Then, the quadrilateral for reication is detected with sub-pixel precision. This enables reication and automatic determination of exposure times during the measurement. This procedure is not possible for 3D objects. Here, the necessary exposure times are selected manually before starting the measurement.

The measurement process is a combination of view-parallel and serialized acquisition. Different inclination angles of the view direction are acquired in parallel. For covering different azimuthal view angles, the sample is rotated into the correct pose by the turntable. We execute our measurement procedure with the goal to minimize the time spent waiting for slow operations to finish. Recording eleven images in parallel takes as long as the maximal exposure time *T* over all cameras plus a constant amount of 375ms for clearing the sensors and transmitting the data. Switching on a light source requires an additional 250 ms delay for the LED to reach stable characteristics. Rotating the turntable by 15° creates an average delay of 9 s. Switching on a projector takes the longest. With the LG projectors we wait for 15 min until the projection stops shifting. In case of the Casio projectors, we wait an average of 60 s until the projector shows the pattern.

For planar samples, we therefore first rotate the turntable. Then, we consecutively cycle through the light sources, always illuminating the sample with exactly one LED, and take an HDR exposure series for each one with all cameras simultaneously. We either use the pre-determined exposure times or employ an automatic exposure compensation. For the auto exposure, we first determine the *region of interest* (ROI) by detecting the registration borders on the sample holder prior to starting the measurement. During measurement, the control computer keeps track of oversaturated and underexposed pixels in the ROI. On that basis, it automatically decides whether additional exposure steps have to be captured. This is done separately for each camera, as the amount of reflected light, and hence the necessary exposure compensation, depends on the camera-angle.

For reconstructing 3D geometry, we also perform a structured light measurement. Here, we first switch on the projector and then rotate through the desired poses. For every rotation we capture a pattern sequence with all cameras. We use a sparser set of 8 azimuthal angles in 45° steps for the geometry acquisition.

Unfortunately, the brightness of the 2.5 W LEDs as well as the brightness of the LED projectors cannot compete with the 575 W lamp of the gonioreflectometer or the flashes of the Dome 1. Hence, the exact measurement time strongly depends on the dynamic range and albedo of the captured material, requiring exposure times up to 5 s or more in disadvantageous cases. To reduce the necessary exposure times, we may perform an electric pre-amplification of the CCD signal, gaining a factor of two. The effective dynamic range of a single image is then reduced to 25 dB, which is still acceptable. In our experiments, acquiring a full BTF measurement took between 4 and 10 h. Additionally capturing the 3D geometry took another 1.5 to 3 h. Typically, we take three differently exposed images for covering a total dynamic range of 60 dB with sufficient overlap. Therefore, the total number of images per measurement amounts to 11 × 3 × 198 × 24 = 156,816 for the appearance and 11 × 3 × 42 × 8 × 4 = 44,352 for the geometry. We found that, given the hardware of the acquisition PC, it is fastest to store the images as 6 MB uncompressed raw data instead of applying an additional compression. Therefore, the total size of the raw measurement data is 918.8 GB for appearance and 259.9 GB for geometry.

## 8. Comparison of Designs

In the previous sections we presented three different setup implementations for the acquisition of digital material appearance. In the course of this, we already highlighted some important differences and similarities as well as advantages and disadvantages. In this section, we will now juxtapose the qualities of our setups together with devices found in the literature. We primarily focus on the different design requirements established in Section 2.3.

### 8.1. Quantitative Comparison Experiments

All three of our setups have generated a fair share of valuable measurements, be it for commercial, scieic or conservation purposes. The UBO20003 and ATRIUM datasets that have been captured with the gonioreflectometer setup have become quite popular for benchmarking and comparing reflectance related tasks, such as compression, fitting or editing. Furthermore with the OBJECTS2011, OBJECTS2012 and SPECTRAL datasets, we recently made several BTFs on 3D objects as well as three full hyper-spectral BTF measurements publicly available to the research community. All of them can be found under http://btf.cs.uni-bonn.de. Our setups have also been used regularly for commercial purposes. Several companies, mostly in the automotive industry, use measured data from our setups for visual prototyping, visualization and marketing. Finally, the Dome 2 setup of Bonn has been placed at the disposal of the Cultural Informatics research group at the University of Brighton in the scope of a one month exhibition in Brighton, UK [92]. Here, the local organizers operated the device on their own and scanned several dozen aacts that were contributed by interested visitors as well as local Cultural Heritage institutions.

In the context of this work, we additionally conducted a series of experiments in order to allow for a quantitative comparison of our setups. We evaluate the following attributes: the achievable dynamic range, the repeatability of a measurement and finally the overall accuracy of the measured reflectance.

First, the dynamic range is assessed in two ways. Since we employ HDR imaging in two of our setups, we report the dynamic range achievable with a single LDR image for each of our measurement instruments and the typical dynamic range that we obtain using HDR photography. In both cases, we consider the strongest radiance *L_h_* that can be observed by the camera without the pixels becoming overexposed and the weakest radiance *L_l_* that can still be distinguished from random sensor noise. The difference *L_h_* − *L_l_* is the maximum detectable interval. Furthermore, we regard the radiance *L_n_* that corresponds to the strength of noise found in a completely black image as the lower bound for the minimal resolvable value (actually any change in value must slightly exceed *L_n_* in order to be distinguishable from random noise). The dynamic range is then expressed in *decibels* (dB) as 10 log_10_((*L_h_* − *L_l_*)/*L_n_*). We make a similar calculation for HDR images that are obtained via exposure bracketing, picking *L_h_* from the image with the lowest exposure and *L_l_* and *L_n_* from the image with the highest exposure. The results are reported in Table 2. The diverse cameras employed in the gonioreflectometer all have a comparably high dynamic range in single LDR images. However, due to the already long measurement times and rapid wear on the involved components, e.g., mirrors of the DSLRs and lifetime of HMI light bulbs, no additional exposure bracketing is employed. This restricts the dynamic range of a gonioreflectometer measurement to the dynamic range of the employed camera's sensor. The cameras of the Dome 1 show a lower dynamic range for single images. Via exposure bracketing this range is increased and surpasses the gonioreflectometer, but is ultimately limited by the available ISO speeds and flash intensity quantities. For single images the Dome 2 has a dynamic range comparable to the Gonioreflectometer. However, here we employ HDR imaging with varying exposure times, measuring almost arbitrarily high dynamic ranges.

We determine the repeatability of the measurement using three criteria. First, we assess the repeatability of the imaging process. This is evaluated by repeatedly switching the cameras off and on, each time taking a picture, and comparing the sub-pixel precise positions of the automatically detected corners of the border markers. We report the standard deviation of the detected corners in pixels. Second, we consider the angular repeatability. We bring all movable components of a setups into a series of configurations that were chosen to reflect the movements during measurement. Then, the respective hardware parts are homed again and the sequence is repeated. Note that due to its completely rigid nature, in the Dome 1 setup only the cameras were turned off and on again. We report the standard deviation of the assumed poses in degrees. In the Dome setups, we capture the sampleholder and find its pose by detecting and triangulating its corners, relying on the geometric camera calibration. For the gonioreflectometer we used Zhang's algorithm [84] to obtain the relative poses of a checkerboard that was mounted on the robot arm. Finally, we also look at the radiometric repeatability by taking a series of pictures of a white-standard under the same illumination condition. During that time, no mechanical movement is carried out. However, in analogy with the reported measurement procedure, the lights of the Dome 2 setup are switched off and on again. We apply the corresponding radiometric correction procedure for each setup and report the remaining variance of the recorded values in percent. All test-sequences were executed 15 to 25 times with all available cameras and for radiometric repeatability of the Dome devices with a selection of 6 different light sources. The resulting figures are reported in Table 2. As expected, the fixed optics of the gonioreflectometer and Dome 2 achieve a good imaging repeatability, whereas the motor-driven lenses of the Dome 1 perform several times worse. Still, the angular repeatability of the Dome 1 by far surpasses the one of the gonioreflectometer due to the otherwise completely rigid nature. Despite the reintroduction of a moving part, *i.e.*, the turntable, the high quality components of the Dome 2 outperform the prior setups in both respects. Concerning the radiometric repeatability, it is apparent that the variation in the flash discharges of the Dome 1 lead to a high deviation, despite the elaborate radiometric correction. The gonioreflectometer performs better, as it uses a a steady light source. Unfortunately, the employed gas discharge lamp is not completely flicker-free. The LED illumination of the Dome 2 provides the best radiometric repeatability.

**Figure 18.**
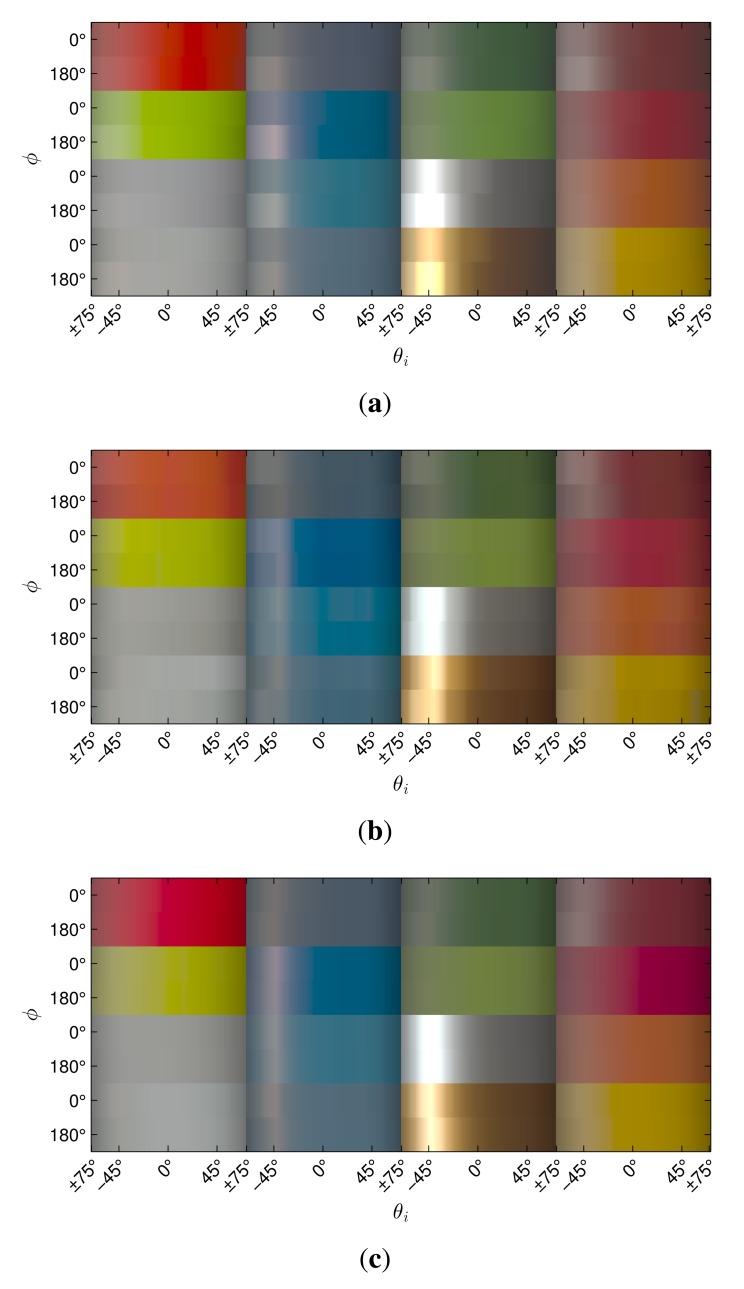
Reflectance measurements of the hand-made material chart (shown in Figure 3). All images depict measurements taken at the fixed camera elevation of *θ_o_* = 45°. The reflectance is visualized as sRGB color values. For the purpose of visualization, the high dynamic range values are tonemapped with a gamma of 3. Measurement values are taken for a semi-circle of illumination directions with *θ_i_* ∈ [−75°, −74°,…, 75° ] under two different azimuthal view and light direction angles *ϕ_i_* = *ϕ_o_* ∈ {0°, 180°}. Each field of the material marker is divided in two rows, corresponding to *ϕ* = 0° and *ϕ* = 180°. The illumination direction *θ_i_* is varied along the *X*-axis. Almost all materials, especially gold and silver, exhibit a specular highlight in the perfect mirror direction at *θ_i_* = −45°. Please refer to Figure 19 for polar plots of the depicted values. (**a**) gonioreflectometer; (**b**) Dome 1; (**c**) Dome 2.

**Figure 19.**
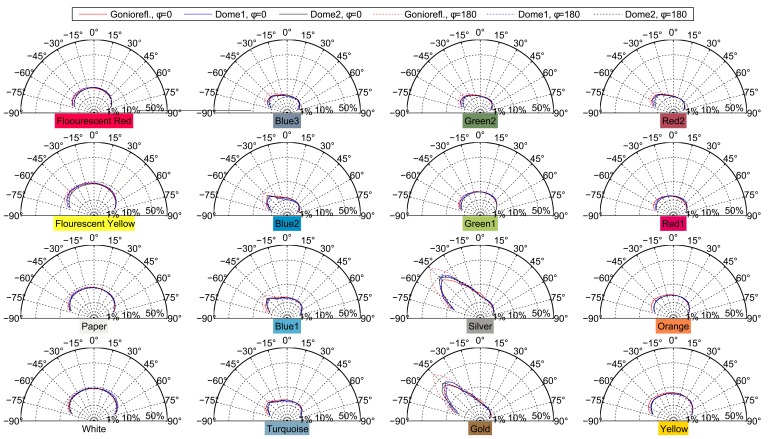
Polar plots of the reflectance distributions shown in Figure 18. The plots show the average reflectivity over all color channels in percent for the respective elevation angles of the light directions. Please refer to the caption of Figure 18 for details about the depicted angles. For the purpose of visualization, the axes are scaled with ∛.

Finally, for determining the overall accuracy, we capture a handmade material chart (first shown in [48]) with all three systems. Figure 3 shows a picture of the prepared chart as well as reied images. To be more robust to small alignment errors, we compute a single average reflectance distribution per material field. We compare the reflectance functions for each field on a set of discrete samples *θ_o_* = 45° × *θ_i_* ∈ [−75°, −74°,…, 75° ] × *ϕ_i_* = *ϕ_o_* ∈ {0°, 180°}. Values for direction combinations that have not been measured are computed via linear interpolation. Note that the obtained reflectance values from the different devices are in no way normalized for this comparison. The only additional operation that is carried out is the conversion of the hyper-spectral measurements of the gonioreflectometer to sRGB, using the CIE 1964 standard colorimetric observer spectrum. The accuracy of each of the setups is assessed by comparing the reflectance functions of the each field for fixed *ϕ_i_* = *ϕ_o_* = 0° with those that have *ϕ_i_* = *ϕ_o_* = 180°. Under the approximation that each material field shows a homogeneous opaque material, both functions should be identical. Deviations between the functions indicate inaccuracies. The results of the experiment are presented in Figures 18 and 19. The gonioreflectometer only meets this condition on rather diffuse materials and reveals inaccuracies in the presence of highlights. The Dome 1 performs better but still shows easily perceivable deviations for many materials. In the measurement of the Dome 2, almost all values are similar (indicated by the two rows in Figure 18c and the black curves in Figure 19 being in good alignment) except in the presence of the very strong specular peak on the gold material.

In Table 2 we provide a comprehensive comparison between our three devices, considering the results of our experiments and the other design attributes.

### 8.2. Comparison with Related Work

**Figure 20.**
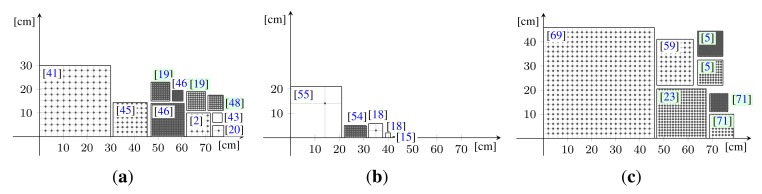
Sampling of the spatial domain for selected setups. Our setups are shaded in green. The size of the rectangles shows the maximum spatial extent of the sampling area. The raster inside the rectangles corresponds to the sampling density with a factor of 1 : 100 on both axes. For [46], [18] and [5], two configurations are shown. Please refer to Table 6 for the exact numbers. Note how in (b) all piecewise planar mirror setups [16,18,55] exhibit a low spatial sampling density and often cover only a small area. (**a**) Gonioreflectometers; (**b**) Mirrors and Kaleidoscope Setups; (**c**) Camera Array Setups.

**Figure 21.**
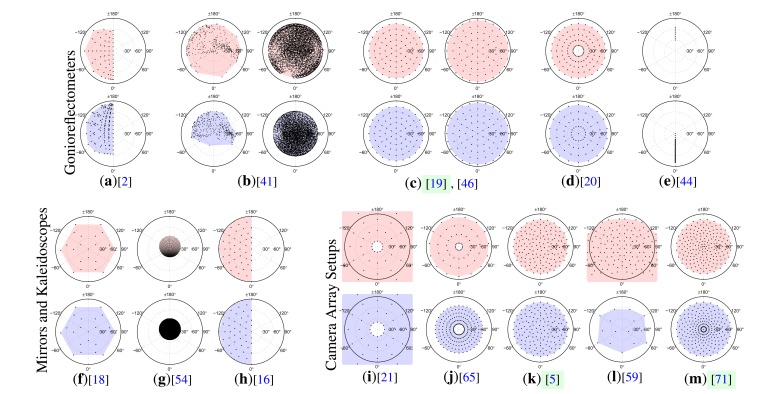
Sampling of the angular domain for selected setups (top disc: light hemisphere; bottom disc: view hemisphere). Our setups are shaded in green. The angle corresponds to *ϕ*, the radius to *θ*. The reported measurement directions *ω* = (*ϕ, θ*) are plotted as black dots, shaded regions emphasize the achieved directional coverage for the sake of easy visual comparison. For (b), directions from the two measurements with fewest and most reported samples are shown. In (c), we illustrate both direction samplings from Table 1. Note that Filip *et al.* [46] adopted the sampling from our setup. The color-shading in (i) and (l) exceeds the plot, because the setups also capture samples at the lower hemisphere. Almost all setups capture the Cartesian product of the indicated view and light directions, *i.e.*, all possible pairs. In contrast, in (a) and (b), each direction participates in exactly one sample. Many setups do only cover parts of the directions on the hemispheres ((a), (b), (e), (g), (h)), have holes ((d), (i), (j)) or show an extremely sparse sampling ((f), (l)). Our setups ((c), (k), (m)) all have a wide direction coverage with densely and equally distributed samples.

The above comparisons nicely underpin the practical value and applicability of our setups. We continue our evaluation by comparing our solutions with the related work found in the literature. Our analysis follows the categorization into the three basic setup classes (gonioreflectometer, mirror and kaleidoscope setups, and camera and light array setups). Note that many publications do not report figures for all design attributes that we described in Section 2.3. This hampers a purely quantitative comparison. We will thus discuss the differences between the reported methods and qualitatively compare the advantages and disadvantages. Still, an overview of the most important common characteristics can be found in Table 6 , Figures 20 and 21.

#### 8.2.1. Gonioreflectometer Setups

Gonioreflectometers are the most common design used for BTF measurement or related reflectance acquisition (SVBRDFs, BRDFs). Hence, there is a large body of work available about these setups, varying in different aspects of the implementation.

As in our gonioreflectometer, several authors propose to have either the light source or the detector at a fixed position and achieve the necessary angular configurations by changing the orientation of the material sample. Most closely related to our approach are the works of Dana *et al.* [2] and Kimachi *et al.* [44], as in both cases the light source position is kept fixed while the material is turned into different poses by a robot arm. In [2], the view direction is changed by manually re-positioning the camera. Due to the manual labor, only a limited set of 7 view directions with different inclination is captured and isotropy of the sample is assumed. Anisotropic materials are considered by measuring them once again with a second azimuthal orientation. In total either 205 or 410 LDR pictures of a material are taken with a video camera in about one or two hours respectively. The authors measured and published a total of 61 BTFs of real-world material samples as part of the CUReT Database (http://www.cs.columbia.edu/CAVE/software/curet/), which was an enormous endeavor and provided valuable datasets. In [44], the camera is re-positioned automatically by a second robot arm. Furthermore, the light source of the setup is equipped with a spectral filter wheel, allowing a multi-spectral acquisition of eight spectral bands. In their experiments, the authors use a sparse sampling with fixed azimuthal angles of *ϕ_i_* = 180° and *ϕ_o_* = 0° and varying inclination angles, amounting to a total of *θ_i_* × *θ_o_* = 8 × 71 = 568 combinations with a higher resolution close to the perfect reflection direction (see Figure 21e). Unfortunately, Kimachi *et al.* do not report too many details about the acquisition process or the data and never seemed to have captured a full BTF, making it hard to judge the other qualities of the setup. Therefore, we do not consider it in Table 6.

A variation of this design is used in the works of McAllister [41], Koudelka *et al.* [20] and Tsuchida *et al.* [42,43]. Here, the camera is placed at a fixed position and the light source is moving. McAllister [41] employs two rotary stages to turn the material sample into different poses and uses a movable arm holding the light source. He reports the capture of 311 to 7,650 angular samples (see Figure 21b) within 45 min to 36 h, being slightly slower than our setup. Because the installed camera did not give access to the raw data, he already accounts for HDR imaging by performing exposure bracketing with up to three different shutter times. In [42,43], a setup with the same principle is extended for hyper-spectral measurement with 16 spectral bands by using a band-pass filter wheel in front of the light source. That setup has been designed for utilization as a desktop device. It has a small extent of 80 cm × 80 cm × 80 cm and is light-weight. As a consequence, the maximum material sample size is restricted to only 4 cm × 4 cm. The authors report the measurement of 6,500 angular samples in 13 h, which is a good result, but only performed BRDF acquisition, not for BTFs. In [20], Koudelka *et al.* mount the light source on a robot arm so it can reach all positions on the hemisphere and the sample is presented to the camera in different angular configurations by a pan-tilt-head. They use a video camera and capture a sampling of 120 × 90 = 10,800 light and view combinations within 10 h (reported in [33]). In their experiments, they acquired a total of 9 material samples. However, the employed video camera captures LDR images with 640 × 480 pixels, yielding a low spatial resolution and dynamic range of the measured data.

Finally, Holroyd *et al.* [45] and Filip *et al.* [46] presented setups that put both, light source and camera, on robot arms. This eliminates the necessity to tilt the sampleholder and hence allows to acquire the same range of delicate materials and 3D objects as leveled mirror setups and camera array setups do. Being the most recent device, the setup proposed by Filip *et al.* [46] in 2013 has a very impressive maximal spatial resolution of 1,071 DPI. This resolution, however, is only achieved for rather small material samples of 4.4 cm × 4.4 cm. The setup uses the same angular sampling of 81 × 81 uniformly distributed directions as our gonioreflectometer and captures RGB HDR data with a full-frame industrial camera. An additional turntable is employed to rotate the sample, as the camera arm only allows movement along one axis. The time needed for acquisition is with 18 h slightly higher than with our setup, possibly because the employed LED light source required longer exposure times. The authors published datasets of six measured materials with 1,071 DPI spatial resolution.

The setup of Holroyd *et al.* [45] has one design detail that is different to all other presented gonioreflectometers: it uses a light source and camera with a beam-splitter on each robot arm to allow a coaxial arrangement of light and view direction. This gives view to the important retro-reflection configuration as well as directly creating reciprocal image pairs. As another advantage, they use self-made fringe projectors as light sources, making it possible to perform structured light reconstruction and utilize high-frequency patterns to separate direct from indirect lighting [93]. Since their setup is primarily intended for 3D object acquisition, they capture a structured light sequence for each view- and light combination, rendering the acquisition process extremely slow. The authors report that they capture 6 × 7 different poses, taking 7 min each. This amounts to about 5 h for as few as 84 angular combinations.

In general, gonioreflectometers offer a great flexibility, as the employed robot arms, tilt-heads or rotation stages can be brought into almost arbitrary angular configurations. Furthermore, the application of only a single light source and a single sensor allows the usage of high-quality components with favorable radiometric attributes, good optics and high resolutions at reasonable costs. Hyper-spectral measurement can be integrated without much effort, using a computer-controlled band-pass filter in front of the light source or sensor [42–44,48].

However, the frequent utilization of moving parts easily introduces inaccuracies. Therefore, a thorough registration of each individual image and calibration of the light and view directions are mandatory. For this, all setups employ additional registration markers next to the sample. Furthermore, while the spatial domain is captured in parallel, the angular configurations have to be measured sequentially, requiring at least one mechanical movement for each. For that reason, the sampling resolution in the angular domain is often considerably lower than in the other two device classes. Measurement times for a sufficient number of view- and light directions vary from 10 [20] to 60 h [48]. To cope with the high number of images shot by a single camera, many setups employ video cameras or industrial machine vision cameras instead of still cameras. However, their sensors often show significantly lower resolutions.

Depending on the design of the gonioreflectometer, measurement of 3D objects as well as easily deformable or hard to fixate material probes, e.g., sand, granules, grass, foliage or fur, may be impossible. Devices that require the sample itself to be rotated into a slope orientation [2,19,20,41–44] cannot be used for these kinds of specimens. However, setups that either employ only a horizontal turntable for the sample [46] or do not move the sample at all [45] could be used for this task (and have so in the latter case).

#### 8.2.2. Mirror and Kaleidoscope Setups

Shortly after the introduction of gonioreflectometers for BTF measurements, setups based on mirrors—either curved or arranged as a kaleidoscope—have been proposed to overcome some of the fundamental shortcomings.

In [52–54], Dana and Wang propose to use a parabolic mirror to capture multiple view directions at once, similar to earlier BRDF measurement setups. To obtain the reflected radiance for different illumination directions, they use a translation stage to move an aperture in the beam of a directional light source. This way, only a small spot on the mirror is lit by the light, which is thereby focused on the material as a cone of illumination with a small solid angle. Furthermore, using a second translation stage, they also move the mirror above the material to capture the spatial variation of the reflectance at different points of the surface. Translation stages offer rather reliable spatial positioning and registration. Thus, Dana and Wang do not employ additional registration markers. Capturing the mirror with a VGA video camera yields the simultaneous acquisition of about 185,500 unique view directions per image. Furthermore, the small iris diaphragm in the aperture covering the light source allows for a generation of about 1,008 unique illuminations. Unfortunately, the employed parabolic mirror design only allows to capture directions between *θ* ≤ 23° and *θ* ≤ 37° elevation (depending on the azimuth angle, see Figure 21g). Hence, reflectance under grazing angles cannot be measured by the device. Furthermore, since the parabolic mirror has a specific point of focus, the device can only capture samples from flat surfaces or geometries with very shallow depths. Handling larger 3D shapes is not possible with this apparatus at all. Despite that, this particular device design suffers from the severe drawback that light directions and spatial dimension again have to be sampled sequentially. In [54], the authors report a sampling of 200 × 200 surface points, which is a rather low number compared to other setups. Although the XY-stage can be moved extremely fast, the acquisition speed is limited by the FPS of the camera and takes about 1 hour per light direction. The authors do not report their exact measurement procedure. However, when conducting a measurement at full extent and highest resolution in all dimensions, the measurement time would amount to 840 days. It is safe to assume that in practice, similar to other sequentially operating devices, a compromise between resolution and acceptable measurement time was found.

Alternatively, a piecewise planar mirror geometry can be employed, with each facet showing the complete material probe from a constant direction. In this case, the spatial domain is captured in parallel as well. Thus a complete outgoing light field is comprised in a single image, allowing a considerable speed-up of the measurement compared to gonioreflectometer devices ([33] reports 1 h for the setup of Han and Perlin [18]). This concept has straightforwardly been employed by Levoy *et al.* [12], Garg *et al.* [13] and Mukaigawa and Tagawa *et al.* [15,16]. In all three cases, planar facets have been arranged in a parabolic or ellipsoidal layout. A digital projector is employed together with a beam splitter to obtain a coaxial arrangement of projector and camera similar to Holroyd *et al.* [45] in Section 8.2.1. Different illumination directions are imposed on the sample by activating only those projector pixels that fall onto one particular mirror-facet. This has the advantage, that all parts of the setup remain fixed, eliminating time-consuming and possibly imprecise mechanical movements. However, similar to the parabolic design of Dana *et al.* [52,53], they only portrait a subset of all directions on the hemisphere, with [16] having the largest coverage (see Figure 21h). We therefore only consider [16] in Table 6. Direct reflections from 50 planar-mirrors are employed, leading to 50 × 50 bi-directional samples.

Han and Perlin [18] and Ihrke *et al.* [55] instead propose kaleidoscope based setups. Here, rather than directly applying many mirror facets on an elliptical shape, a clever arrangement of three planar mirrors provides a set of recursive interreflections that provide a multitude of virtual viewpoints at once to the camera. Again, digital projectors are used for creating different light directions. There is no mechanical movement in these setups. In [18], two different angles of taper have been explored, forming 22 × 22 and 79 × 79 direction combinations, respectively. Figure 21f demonstrates the obtained direction sampling with 22 directions. Ihrke *et al.* [55] use their setup to capture 3D objects instead of flat material samples. They employ an additional mirror below the object that gives view to the lower hemisphere. This way they obtain 246 virtual views and 144 virtual light sources, yielding 35,424 direction-pairs distributed over the full sphere. Note that for flat samples only those pairs that lie in the upper hemisphere can be considered, reducing the number of useful direction combinations to 8,856.

Similar to gonioreflectometers, the fact that only one camera and one light source (a projector) is necessary facilitates the usage of high-quality components. Hyper-spectral measurements should be directly possible as well, but this has not yet been topic of active research. However, a single camera also means that four measurement dimensions (*ω_o_* and x) are embedded into the same two dimensional space on the sensor. This implies a trade-off between spatial resolution and the number of possible direction combinations. Bangay and Radloff provide a detailed analysis of this issue for kaleidoscopic configurations [94]. Furthermore, similar to some camera arrays, such as our Dome 1 setup, the resolution of the angular dimensions is ultimately fixed during construction time. The spatial resolution therefore directly depends on the resolution of the employed sensor. Han and Perlin [18] use 3.1 Megapixels and achieve about 65 pixels for a sample size of 19 mm (for the 79 × 79 sampling). Mukaigawa and Tagawa *et al.* [15,16] report a similar number of 60pixels for a sample size of 6mm using a 5 Megapixel sensor. The resolution of the employed projector is a minor detail, since in principle—for BTF measurements—even a single pixel per mirror-facet would suffice.

The usage of a camera and a projector suggests a possible application of structured light for 3D reconstruction. While the coaxial arrangement of light source and camera does not provide the necessary stereo basis for triangulation, the multiple virtual viewpoints formed by the interreflections could be used instead. Still, a multi-view triangulation is complicated by the fact that a 3D object will occlude parts of the mirrors and hence overlay the image in the virtual view points. It therefore depends on the unknown shape of the object which parts of an image depict the object from the correct perspective. So far, only Ihrke *et al.* [55] tackled this problem, eventually reconstructing geometry and surface reflectance using a kaleidoscope.

The piecewise planar mirror based setups [12,13,16,18,55] do not employ any moving parts, making it possible to establish a registration of the data a-priori and thus avoid auxiliary registration markers. Yet, a precise calibration poses a more severe problem than for gonioreflectometers or camera arrays, as at least one if not several levels of indirection due to interreflections have to be considered. In [95], Isaac Newton observed *“[*…*], that every irregularity in a reflecting superficies make the rays stray 5 or 6 times more out of their due course, than the like irregularities in a refracting one”* (pp. 3079–3080), arguing that mirror based optics have to be manufactured with much higher precision than lens based ones to achieve similar accuracy.

This is also one of the reasons why we opted against implementing a piecewise planar mirror based setup on our own and rather explored two camera array devices. The second reason can be found in the fact that all presented mirror based setups exhibit either an extremely restricted measurement volume, a very low spatial resolution or both (see Figure 20b). Still, the number of sampled directions is often similar or worse than what our gonioreflectometer was able to obtain in an acceptable amount of time (compare Figure 21f,h to Figure 21c). Of course, employing higher resolution sensors would directly increase the spatial resolution while measurement times would stay constant. Similarly, the measurement volume can be increased using larger mirrors. But then again, both modifications would further raise concerns about accuracy. The apparatus proposed by Dana and Wang [52–54] does in theory not show these tight restrictions. However, their approach only captures a small portion of the hemispherical directions and comes at the cost of impractically long measurement times.

#### 8.2.3. Camera and Light Array Setups

Consequently, setups based on camera arrays follow a different avenue to cope with the acquisition time drawback of the gonioreflectometer. Using multiple cameras, parts of or even the full outgoing light field are captured in parallel without sacrificing sensor resolution or accuracy. Lenses with different focal lengths make camera arrays flexible with respect to resolution and measurement volume.

Debevec *et al.* [10] proposed a setup, introduced as “Light Stage”, that utilized two cameras with fixed positions together with a light source mounted on a two-axes rotation system to capture reflectance fields of human faces. The setup was later extended to utilize a rotating arc with an array of 27 lights [56] and eventually a fixed dome with 156 light sources [57,58]. The authors aim for real-time acquisition of reflectance data. For this, they utilize costly high-speed cameras, capturing all possible samples within few seconds (60 s [10], 15 s [56], 83 ms [58]). The high price of these cameras leads to an insufficient sampling of the outgoing light field with only one or two view directions. We therefore disregard the Light Stage setups in our further comparison. Weyrich *et al.* [59,60] follow up on the last approach by Debevec and Wenger [57,58] and present a system with 150 light sources evenly distributed on a geodesic dome. Here, however, 16 cameras are employed to simultaneously capture the reflectance samples, leading to a slightly better coverage of the view direction domain (see Figure 21l). Nonetheless, this approach still shows an insufficiently low view direction sampling for purely data-driven bi-directional material representations. Recently, Hu *et al.* [62,63] proposed a design very similar to Weyrich's [59]. They employ a geodesic dome with twelve cameras and 238 light sources. However, their cameras are all mounted on a single vertical arc. Therefore, all cameras lie on a single azimuthal angle, eventually sampling a 5D slice of the BTF. Yet, as the cameras are arranged at different inclination angles, this still adequately captures isotropic reflection. Wu *et al.* [61] also present a system that captures only a 5D slice of the BTF. Their design has a horizontal ring of 20 cameras and a dome of 290 LED light sources. In contrast to the setup of Hu *et al.* , this 5D slice does not correspond to a meaningful subset of the reflectance function, as the cameras are all arranged on a single inclination angle.

In [21], Furukawa *et al.* employ five cameras and six light sources, equidistantly installed on two separate vertical arcs. The sample is placed on a rotation stage at the center and the arc containing the light sources can be horizontally rotated as well. This way, the full sphere is covered for both directional domains with 72 × 60 samples. For flat materials this number would be reduced, as view and light directions have to be located on the upper hemisphere in this case, yielding 36 × 36 samples (see Figure 21i). Similar to our Dome 1 design, Furukawa *et al.* employ point-and-shoot cameras. Tong *et al.* [67] use the very same principle with eight industry cameras and eight light sources. They capture BTF data with a comparable angular sampling as our gonioreflectometer in two hours. Matusik *et al.* [64,65] employ a similar setup design as well. They use four light sources and six cameras and additionally capture matting images and make use of two computer-screens, placed below and behind the sample, to sample light that is transmitted through the surface more densely. In our comparison, we only consider the set of view and direct light directions that would be used for BTF capture. Here, they acquire 60 × 216 (= 12,960) angular samples. Although the total amount of combinations lies between our gonioreflectometer and the Dome 1, the set of directions is not as well-balanced, putting a considerably higher emphasis on the view domain. Due to the large amount of mechanical movement (at least 540 operations), Matusik *et al.* require about 14 h to capture all samples, despite the use of industrial video cameras. Similar to our Dome 2 setup, Matusik *et al.* employ two sets of prime lenses to account for objects of different size. Unfortunately, none of the three papers reports on measurement volume and achievable resolution.

Recently, Köhler *et al.* [69] and Nöll *et al.* [70] presented a setup called “OrCam”, where an array of seven cameras that can be rotated to acquire different inclination angles is combined with a spherical gantry mounting a total of 633 light sources at fixed equidistant positions. Similar to [21,64,65,67], the sample is placed on a turntable to capture different azimuthal angles. They report to capture 133 different view directions. The LED light sources are combined to a total of 19 illumination patterns per view, resulting in 2,527 combinations. In contrast to the Dome 1 and Dome 2, their setup is explicitly designed to capture large 3D objects. It has a larger diameter and uses wide-angle lenses. This way, the authors can cover a measurement volume with a diameter of 80 cm with a moderate spatial resolution of about 127 DPI. With a measurement time of up to 1.2 h, the OrCam takes about three times longer per direction than the Dome 2. Similar in spirit, Neubeck *et al.* [66,68] present the “KULETH Dome”. It utilizes a rotation-stage together with a single camera on a robot tilt arm to capture different viewing directions. They also employ a dome of 169 affixed light sources, in this case a quarter-sphere, to sample the different illumination directions. However, using only one camera this setup does not offer the advantage of simultaneous acquisition but only benefits from the reduced mechanical effort in sampling the different light directions.

Setups that make use of camera and light arrays have the potential to gain a considerable speedup compared to sequential gonioreflectometers without the necessity to trade-off spatial with angular resolution as mirror based setups do. Three devices attempt to capture all view directions on the full hemisphere simultaneously and abandon any moving parts: Our Dome 1 setup [5] as well as the setups of Weyrich *et al.* [59,60] and Hu *et al.* [62,63]. Not surprisingly, those devices are also the fastest camera array setups and among the fastest BTF capturing setups in general. [59] capture 2,400 images with 1.3 Megapixels in only 25 s. However, Weyrich and Hu only have a sparse set of directions (16 and 12 cameras, respectively) covering the view hemisphere, whereas our Dome 1 setup provides as much as 151 directions. Unfortunately, the necessary amount of cameras to densely cover the full view direction hemisphere leads to increased costs as well as control and synchronization issues. Therefore, many camera array devices instead follow a hybrid approach, combining a smaller arrangement of cameras with a turntable [21,64,65,67,71] and sometimes also an additionally movable tilt arm [66,69,70]. Yet, in these cases the need for additional sequential capture in the view domain leads to an increase in measurement time.

Due to the reduction of mechanical sample movement to at most a rotation of a turntable as well as the usually large dimension to host all hardware-parts, camera and light array setups lend themselves for the acquisition of reflectance from 3D objects. Almost all presented setups with the exception of [62,63,66–68] have therefore reported their successful application for this task:

In [21,22,64,65] the silhouette of the object is extracted in the images for each view direction. The visual hull is constructed via volume carving [96] or using an image based visual hull technique [97]. This approach has the advantage that the reconstructed geometry is correctly aligned with the captured images for different view directions. Unfortunately, the visual hull cannot reconstruct concavities correctly. Furthermore, inaccuracies in the silhouette extraction can lead to rather crude approximations of the actual shape. To improve upon these drawbacks, in [21] an additional laser range scanner is employed. Yet, in turn, this requires to register the 3D geometry obtained by the laser range scanner with the appearance measurement. Similar, in [59,60] an auxiliary structured light based 3D scanner is employed. Here, feature-points are matched between the scanner-generated texture data and the images of the reflectance measurement. The geometry captured from the separate scanner is registered to the cameras using these correspondences.

More recent devices [23,69–71,74] instead employ an integrated structured light based 3D reconstruction, using the same views that are used for reflectance acquisition to capture the 3D surface via triangulation. This way, the geometry is already registered with the reflectance measurements, at the expense of requiring accessory fringe-projectors in the setup. In [83], it is shown that the combination of multiple fringe projectors and the multi-view design of reflectance measurement devices can be used obtain a very accurate and dense sampling of the surface geometry. Consecutive work [73] incorporates reciprocity of view and light directions, encountered in many of the setups, into the 3D reconstruction as well, by the means of a volumetric probability distribution.

## 9. Conclusion

In this work, we have ideied a list of basic attributes that should be fulfilled by BTF capturing devices. Subsequently, we surveyed the literature for existing approaches that meet the established requirements and found that these setups can be categorized in three primary device classes: Gonioreflectometers, mirror based setups and camera array setups. Each of the classes has its distinct advantages and drawbacks. We illustrated this by discussing one gonioreflectometer and two different camera array designs in great detail. Furthermore, we compared them with each other with respect to all of the ideied attributes. Finally, we also took the approaches from the surveyed literature into account and pointed out similarities as well as unique solutions found in the variety of proposed setups.

In the end, there is no single device that outperforms the others on all disciplines. There is not even a clear tendency towards one of the main device classes. Instead, different approaches focus on different aspects of the BTF acquisition.

We believe that our most recent Dome 2 setup provides state-of-the-art performance and a well balanced compromise between many of the practical aspects. However, which device class or particular setup design is best suited depends on the application at hand. The presented comparison of the basic attributes in Table 6 can be an aid for decision-making. Still, it is hard to grasp the practical applicability of many of the setups, as very little is reported on the topics of reliability, durability, *etc.* In this case, the in-depth discussion of our three implemented devices can serve as an indicator what problems can be expected, which device class handles them best and and how much effort is necessary to tackle them.

### 9.1. Lessons Learned

Many considerations for an acquisition setup depend on its intended application. For pursuing truly general appearance capture on a larger scale than capturing a handful of samples with a laboratory prototype, we would recommend the following:

- Use steady light sources. While strobe light sources might provide a good photon yield and avoid unnecessary exposure, they enormously complicate the radiometric calibration, which eventually leads to increased effort for every single measurement and probably reduced repeatability and accuracy.
- Avoid mechanical movement whenever possible. This improves measurement speed as well as reliability and accuracy. The fastest capture setups in the literature follow exactly this strategy. Mechanical movement is also one of the reasons why our Dome 2 setup lacks the speed of its predecessor.
- Do not use point-and-shoot cameras or similar consumer grade devices, such as smart-phones. Those devices usually require a lot of compromises and perform unavoidable unwanted operations. Long transmission times, missing raw capture support, “image improvements”, bad repeatability and necessity for using the auto-focus are just a few of the drawbacks we encountered in our Dome 1 setup. Furthermore, although the nominal spatial resolution of the Dome 1 setup is higher, images taken with the Dome 2 are still sharper, because of the better optics and the access to raw images without JPEG compression aacts.
- Use sufficiently strong light sources. Comparably weak LED light sources are the major reason why our Dome 2 setup is far beyond its capture frame-rate potential. In the case of the spectral gonioreflectometer setup even 575 W are not enough. Due to the narrow spectral band filtering, long exposure times of several seconds per image can be necessary.
- The camera array plus light dome design is probably the way to go. It has recently been adopted by other groups as well (e.g., [59,62,66,69]) and view-parallel acquisition seems to be the most promising approach to keep measurement times in balance for capturing a high number of direction samples.
- For larger camera arrays, plan a distributed acquisition setup with a client-server architecture and sufficiently many camera control computers. To avoid the bottleneck of transmission with USB 2.0, we equipped our control computers with additional USB 2.0 PCI cards. However, we found that the PCs could not handle more than 20 simultaneously connected cameras without occasional hiccups. Although we did not yet reach a similar limit with the 13 Gigabit-Ethernet connections in the Dome 2 setup, the throughput of the employed bus system (Dome 1: PCI; Dome 2: PCIe) will at some point become a bottleneck as well.
- Consider the trade-off between bandwidth (and storage requirements) and CPU load. In the Dome 1 setup, the cameras' internal processor applied a JPEG compression, allowing the transmission and storage of up to 19 images with 12 Megapixel within a few seconds and without significant load on the control computer. However, the Dome 2 cameras deliver a raw data stream. Here, we found ourselves in a dilemma: On the one hand, a too elaborate compressed image format (e.g., OpenEXR (www.openexr.com)) would reduce the image throughput due to limited CPU capabilities, even with two fast Intel Xeon processors. On the other hand, directly storing the RAW image data lets the hard disk's write speed become a considerable bottleneck. We eventually employ a very lightweight self-written lossless image compression but are still occasionally limited by the disk speed.

### 9.2. Limitations & Future Work

There are still several limitations and possibilities for future work that can be found throughout the entire spectrum of existing methods.

The time requirement for a single measurement is still a limiting factor for the wide-spread application of BTF measurements. Our Dome 2 setup demonstrates that one main concern are prolonged exposure times due to the low amount of reflected radiance for many directions. A simple solution to this problem is to use more powerful light sources. For instance, the setup of Weyrich *et al.* [59] uses 103 LED emitters per lamp to provide sufficient illumination for capturing the reflectance at 12FPS. However, this compromises the assumption that the light at each point is coming from a single direction. Furthermore, it can severely impact costs in case a light dome is employed. Another possibility to improve the amount of irradiance during measurement is the acquisition with illumination from multiple light directions at the same time. Using an appropriate set of illumination patterns, the appearance under a single light source can later be reconstructed by solving a linear equation. The “LightStage 5” setup in [58] already implements this idea.

All three of our setups, as well as almost all of the discussed setups in the related work, are bulky laboratory devices. Our Dome 2 setup and the OrCam [69] begin to approach on-site usability by being dis-mountable into separate parts that can be transported. Tsuchida *et al.* [42] already explicitly addressed this matter by proposing a table-top design with 80 cm × 80 cm × 80 cm. However, there are also other examples for more compact and transportable setups, such as a table-top single-view light-dome [98] that fits into a briefcase or an SVBRDF acquisition tool-set that fits in a pocket [99]. Yet, it will require further research until fast and comprehensive appearance measurement becomes applicable directly on the desks of designers or in easily deployable tools for digitization professionals in cultural heritage or other industries.

Another limitation, common to almost all discussed setups, is the sampling resolution in the angular domains. Whereas the prevalent high spatial resolution of 200 DPI to 450 DPI with 10^5^ to 10^6^ of sampling points per material sample is sufficiently dense to provide a continuous impression of the material's surface, the highest complete angular resolution that we found is 198 × 264 directions for the view and light domain in our Dome 2 setup, *i.e.*, a resolution of about 8° and 9°, respectively. However, a study about data-driven BRDF models [100] shows that preserving the highlight of specular materials requires resolutions considerably below 1°. Unfortunately, their approach to utilize a denser sampling close to the highlight is not applicable for measurement from inhomogeneous surfaces as the highlight direction might be different in every single point. Ruiters and Klein [101] argue that a shift in paradigm away from capturing discrete samples towards measuring weighted integrals might help to solve this problem. Thus, the utilization of spatially extended pattern illumination (see [102,103] for two recent SVBRDF approaches) for BTF measurement would be an interesting avenue of future research.

Ultimately, the discussed measurement setups can only provide data for BTFs and thus mostly opaque materials. For strongly globally subsurface-scattering, translucent or completely transparent materials or objects, a new class of BSSRDF measurement devices would be required. Some of the presented setups [59,60,67] tackle this problem using an auxiliary measurement for subsurface scattering approximation. Existing experimental setups for full BSSRDF measurement [12–16] only capture fractions of the angular domains. Moreover, none of the setups considers the wavelength and time dependent redistribution of energy, found in the full 12 dimensional scattering function *S* (Equation (1)). It thus remains a challenging problem of future research, how to effectively sample such a high-dimensional appearance space within reasonable acquisition times and disk-space requirements.

## Figures and Tables

**Table 1.** The sampling of the hemisphere used during a measurement with our gonioreflectometer setup. View and illumination hemispheres are sampled identically, but two different sets of directions have been employed, depending on the material. *: only one direction at *ϕ* = 0°.

*θ*_1_	Δ*ϕ*_1_	*θ*_2_	Δ*ϕ*_2_	# dir	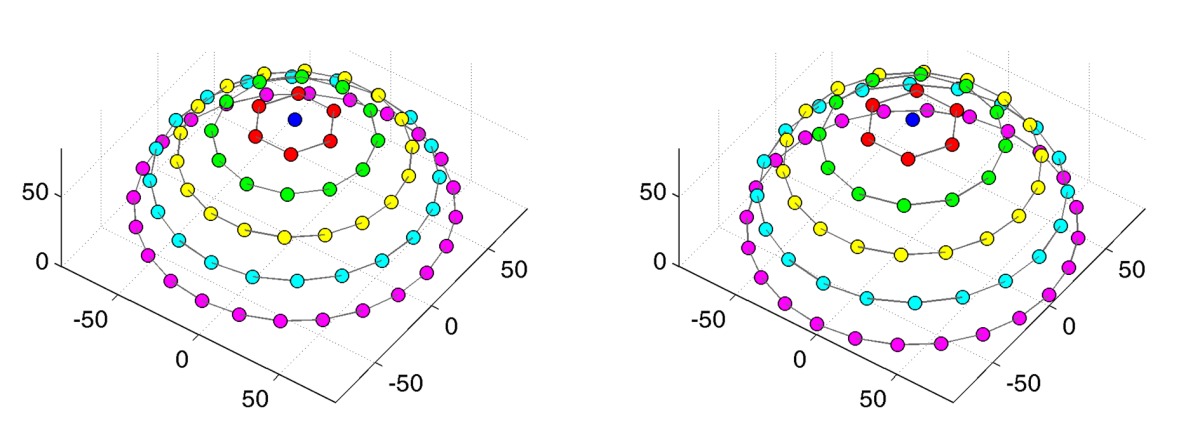

0°	_*	0°	_*	1	
17°	60°	15°	60°	6	
34°	30°	30°	30°	12	
51°	20°	45°	20°	18	
68°	18°	60°	18°	20	
85°	15°	75°	15°	24	

**Table 2.** Comparison of our setups with respect to the design requirements.

	**Gonioreflectometer**			**Dome 1**			**Dome 2**
configuration	2002 [19]	2004 [5]	2010 [48]	2004 [5]	2008	2011 [23]	2012 [74]
dimensions (L×W×H) [cm]	410 × 170 × 90			190 × 190 × 190			340 × 250 × 250
distance to sample [cm]	170 / 240			65			100
directions *ω_i_* × *ω_o_*	81 × 81			151 × 151			198 × 264
resolution *ω_i_*	14.7° ± 0.4° / 16° ± 0.8°			9.4° ± 1°			9° ±1.2°
resolution *ω_o_*	14.7° ± 0.4° / 16° ± 0.8°			9.4° ± 1°			7.6° ± 2.6°
maximum *θ*	75°/85°			75°			75°
focal length^11^ [mm]	240	180	270	116	104	52 – 104	95 / 190
spatial resolution [DPI]	280	330	290	235	450	225 – 450	190 / 380
dynamic range^1^ [dB]	35/-/∞	31/-/∞	32/-/∞	28/33/33	25/44/44		32/60/∞
spectral bands	RGB		32	RGB			RGB
camera type	DSLR		Industrial	P&S			Industrial
camera data	12 BPP raw			8 BPP JPEG			12 BPP raw
light source type	gas discharge lamp			flash			LED
measurement volume [cm]	8^2^		6.5^2^	10.5^2^	10.5^2^	10.5^3^−20.5^3^	7.5^2^ / 24^3^
direction variation	1.9° / 1.4°		1.5° / 1.1°	6.5°		7.8°−18°	3°/12.6°
BTF raw images	6,561		209,952	91,204			156,816
BTF time [h]	14		60	1.8			4.4−9.7
BTF size [GB]	83		1,228	22	281		918
3D raw images						171,234	44,352
3D time [h]						1.4	1.5–3
3D size [GB]						282	260
radiometric repeatability^2^	-^10^		1.1%_0_	-^10^	7.4%_0_		0.1%_0_
geometric repeatability^3^	-^10^		0.17 px/0.61°	-^10^	0.81 px/0.006°		0.12 px/0.002°
sampling flexibility	full: arbitrary *ω_i_* and *ω_o_*			none			some: arbitrary *ϕ_o_*
radiometric calib. procedure	easy			complex			easy
geometric calib. procedure	manual			automatic			automatic
durability (# measurements)	> 12^4^	≈ 27^5^	> 1000^6^	–^7^	≈ 265 / > 347^8^		> 3650^9^

1. Single exposure/performed HDR measurements/theoretical max0imum;

2. Given as variance in measured reflectivity for SphereOptics Zenith UltraWhite;

3. Standard deviation in imaging condition in pixels/Standard deviation in angular configuration in degrees;

4. Camera use discontinued at about 83,000 exposures. Probably limited by wear on mirror;

5. Defect of mirror at about 180,000 exposures;

6. Assuming one measurement every three days and continuous camera operation for 10 years. Note that the HMI bulb has a lifetime of 1,000 h and therefore has to be replaced about every 16 measurements;

7. Not determined due to systematic defect of the CCD chips in the whole camera series [82];

8. Two camera CCDs became defective and were replaced after about 160,000 exposures. The other 149 are counting 210,000 exposures and probably limited by the wear of the flashes;

9. Assuming one measurement per day. The camera manufacturer asserts continuous operation for at least 10 years. Tests with the LEDs indicate a lifetime of at least 4,000 measurements;

10. The repeatability tests were omitted for outdated configurations;

11. 35 mm equivalent focal length.

**Table 3.** The fixed hemispherical direction-sampling in our Dome 1 setup. *: only one direction at *ϕ* = 0°.

***θ***	**Δ*ϕ***	#**dir's**	

0°	_*	1	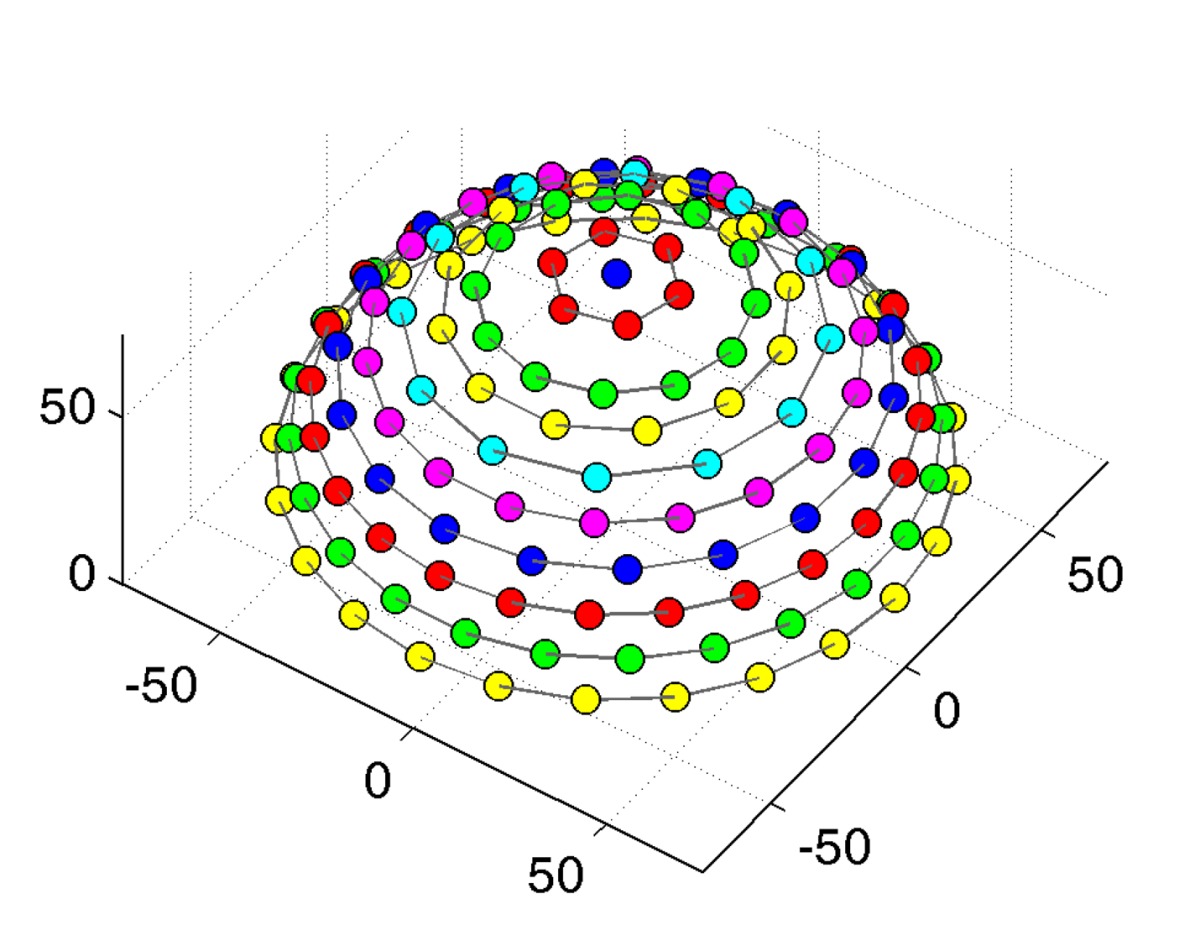
11°	60°	6	
23.5°	30°	12	
30°	30°	12	
37.5°	30°	12	
45°	20°	18	
52.5°	20°	18	
60°	15°	24	
67.5°	15°	24	
75°	15°	24	

**Table 4.** Overview of symbols and terms used in the radiometric calibration of the Dome 1.

*c*	shooting camera index in [1, 2,… ,151]
*f*	flashing camera index in [1,2,… ,151]
*i*	ISO speed setting in {50,100, 400} for PowerShot A75 and {80,100, 400} for PowerShot G9
*q*	flash intensity quantity in {minimum, medium, maximum}
r	a specific combination of employed flashing camera, flash quantity and ISO speed, *i.e.*, a tuple r = (*f, q, i*)
*I^c^*^,r^	image of camera *c* taken under radiometric conditions r
$I_{\text{x},\lambda }^{c,\text{r}}$	8- bit pixel value of image *I^c^*,r for spatial position x and color-channel λ
λ	color-channel index in {red, green, blue}
*χ*_*c,i*,λ_	response function of camera *c* for ISO speed *i* and color-channel λ
$L_{\text{x},\lambda }^{c}$	radiance (in Wm^−2^sr^−1^) for spatial position x and corresponding direction towards camera *c* for color-channel λ
*k*	index of radiometric calibration marker in {1, 2, 3, 4}
*a_k_*	albedo of radiometric calibration marker *k*
*m̄*_r,*k*_	average pixel value for marker *k* in the image of camera 1 under radiometric condition r
*Ê*_r,*k*_	predicted irradiance (in Wm^−2^) at marker *k* for flash discharge with attributes r
v*_f,k_*	vector in ℝ^3^ from marker *k* to flash *f*
‖v*_f,k_*‖_2_	distance between marker *k* and flash *f*
n*_k_*	surface normal in ℝ^3^ of marker *k* with ‖n*_k_*‖_2_ = 1.
n*_f_*	central direction in ℝ^3^ of the flash cone for flash *f* with ‖ n*_f_* ‖ _2_ = 1. (identical to the orientation of the camera with index *f*)
*w*	continuous weighting function with *w*(*p*) = 0 for underexposed or saturated and *w*(*p*) = 1 for well exposed values *p*
*Ê*_r,x_	predicted irradiance (in Wm^−2^) at spatial position x for flash discharge with attributes r
*E*_r,x_	true irradiance (in Wm^−2^) at spatial position x for flash discharge with attributes r

**Table 5.** The hemispherical direction-samplings in our Dome 2 setup. On every second ring, the azimuthal view angle *ϕ_o_* is displaced by *φ_o_* = 7.5°. On each ring, the azimuthal light angle *ϕ_i_* is displaced by *φ_i_* to be arranged symmetrically around the cameras. Furthermore, there is one additional lamp in every ring except the first at *ϕ_i_* = *ϕ_o_* + 180°, *i.e.*, the perfect mirror direction of the respective camera.

264 view directions				198 light directions				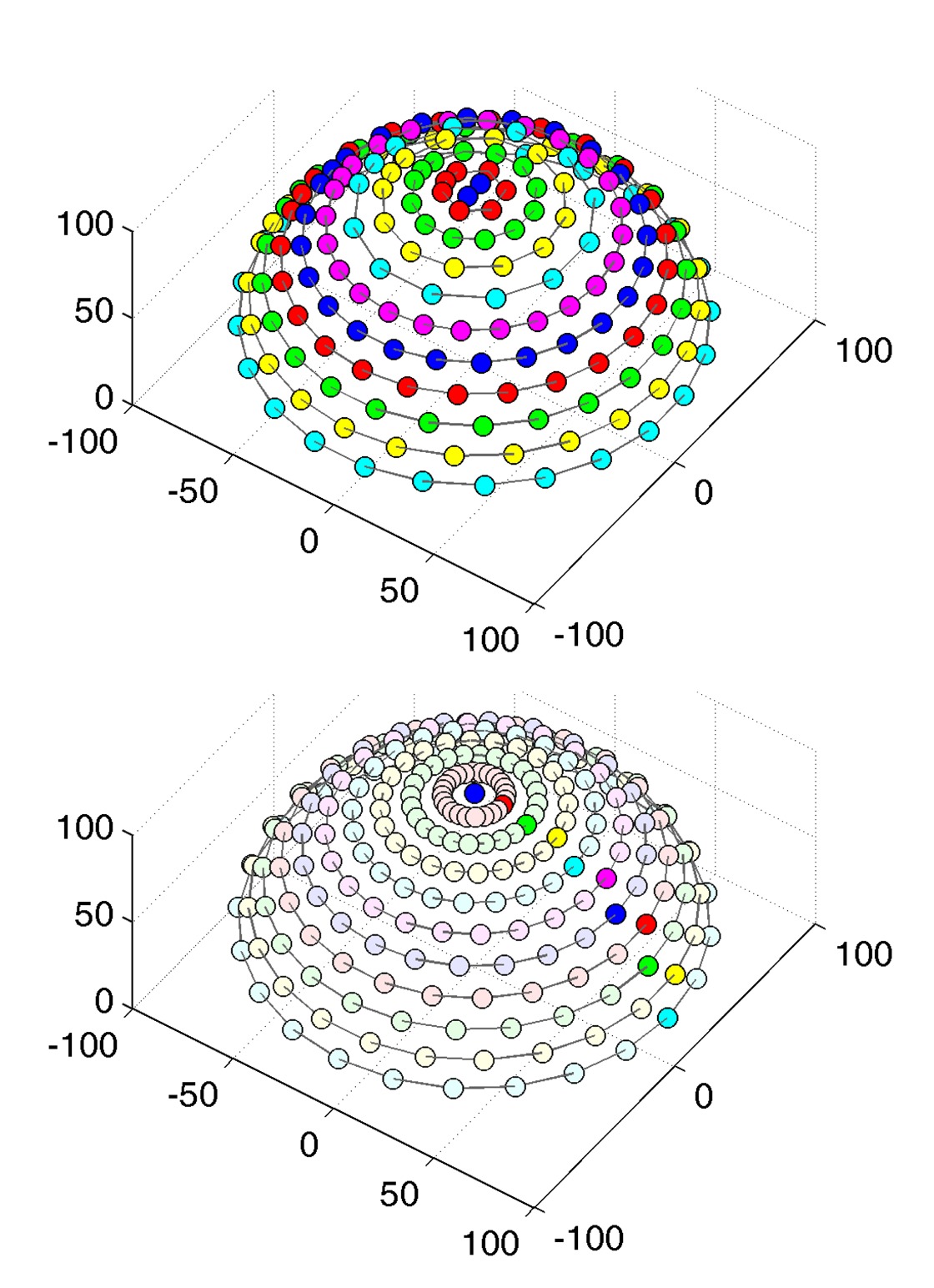
*θ_o_*	Δ*ϕ_o_*	+ *φ_o_*	#dir's	*θ_i_*	Δ*ϕ_i_*	+ *φ_i_*	#dir's	
	
0°	15°	0°	24	2.5°	180°	90°	2	
7.5°	15°	7.5°	24	7.5°	60°	37.5°	6 + 1	
15°	15°	0°	24	15°	30°	15°	12 + 1	
22.5°	15°	7.5°	24	22.5°	30°	22.5°	12 + 1	
30°	15°	0°	24	30°	30°	15°	12 + 1	
37.5°	15°	7.5°	24	37.5°	15°	0°	24+1	
45°	15°	0°	24	45°	15°	7.5°	24+1	
52.5°	15°	7.5°	24	52.5°	15°	0°	24+1	
60°	15°	0°	24	60°	15°	7.5°	24 + 1	
67.5°	15°	7.5°	24	67.5°	15°	0°	24+1	
75°	15°	0°	24	75°	15°	7.5°	24+1	

**Table 6.** Comparison with other setups. Our setups are shaded in green. The numbers of other setups are compiled from publicly available sources: the cited original research publications, associated technical reports, state-of-the-art reports and courses covering the devices as well as websites of accompanying databases or laboratories. Some figures have not been directly reported and are instead derived from the available material (see footnotes). GDL stands for gas discharge lamp.

publication	year of publication	sample size [cm^2^]	spatial resolution [DPI]	# direction samples	measurement time [h]	Speed^10^ [Megasamples/s]	# cameras	camera type	# light sources	light source type	HDR capture	# spectral bands	3D objects

Gonioreflectometers													
[2] Dana *et al.*	1997	10 × 10	114	205^1^	1	1.4	1	Video	1	GDL	**✗**	3	**✗**
				410	2								
[41] McAllister	2002	30 × 30	100	311	0.75	20	1	DSLR	1	GDL	**✓**	3	**✗**
				7,650	36	10							
[19] Sattler *et al.*	2002	8 × 8	280	6,561	14	4.9	1	DSLR	1	GDL	**✓**	3	**✗**
	2004		330			6.8							
[20] Koudelka *et al.*	2003	4.7 × 4.7^2^	100	10,800	10	1.3	1	Video	1	LED	**✗**	3	**✗**
[43] Tsuchida *et al.*	2005	4 × 4	?	6,500	13	?	1	Industrial	1	GDL	**✓**	16	**✗**
[48] Rump *et al.*	2010	6.5 × 6.5	290	6,561	60	22.3	1	Industrial	1	GDL	**✓**	32	**✗**
[45] Holroyd *et al.*	2010	14.4 × 14.4^3^	127	84	5	0.3^11^	2	Industrial	2	Projector	**✓**	3	**✓**
[46] Filip *et al.*	2013	4.4 × 4.4	1,071	6,561	18	43.5	1	Industrial	1	LED	**✓**	3	**✗**
		14 × 14	350			47.1							

Mirror and Kaleidoscope Setups													

[18] Han and Perlin	2003	5.8 × 5.8^4^	85^4^	484	1	0.6	1	P&S	1	Projector	**✗**	3	**✗**
		2 × 2^4^		6,241		1.0							
[54] Dana and Wang	2006	9 × 5	339	1.9·10^8^ ^5^	20,160^6^	262	1	Industrial	1	Tungsten	**✗**	3	**✗**
[16] Mukaigawa *et al.*	2010	0.6 × 0.6	250	2,500	?	?	1	Industrial	1	Projector	?	3	**✗**
[55] Ihrke *et al.*	2012	21 × 21^3^	18	35,424	93.5	0.3^12^	1	DSLR	1	Projector	**✓**	3	**✓**

Camera Array Setups													

[21] FFurukawa *et al.*	2002	?	?	4,320	?	?	5	P&S	6	Tungsten	**✗**	3	**✓**
[65] Matusik *et al.*	2002	?	?	12,960	14	?	6	Industrial	4	GDL	**✓**	3	**✓**
[5] Müller *et al.*	2004	10.5 × 10.5	235	22,801	1.8	414	151	P&S	151	Flash	**✓**	3	**✗**
	2008		450			1,520							
[59] Weyrich *et al.*	2005	15.2 × 19.1^8^	130^9^	2,400	0.007	9,044	16	Industrial	150	LED	**✓**	3	**✓**
[66] Neubeck *et al.*	2005	?	230^7^	44,616	?	?	1	?	169	?	**✗**	3	**✓**
[67] Tong *et al.*	2005	?	?	7,056	2	?	8	Industrial	8	Tungsten	**✓**	3	**✗**
[62] Hu *et al.*	2010	?	?	2,856^1^	0.5	?	12	?	238	LED	**✓**	3	**✗**
[23] Schwartz *et al.*	2011	20.5 × 20.5^3^	225	22,801	3	1,617^13^	151	P&S	151	Flash	**✓**	3	**✓**
		10.5 × 10.5^3^	450			1,696^13^							
[71] Schwartz *et al.*	2013	10 × 10	190	52,272	4 –10	132 ^13^	11	Industrial	198	LED	**✓**	3	**✓**
		7.5 × 7.5	380			297 ^13^							
[69] Köhler *et al.*	2013	46 × 46^3^	127	2,527	0.7–1.2	493 ^12^^,^^13^	7	DSLR	633	LED	?	3	**✓**

1 Only isotropic reflectance sampling;

2 Estimated from the Lego-brick sample, depicting 6 × 6 nobs;

3 Footprint of reported measurement volume on the ground plane;

4 Estimated using the size of a penny coin depicted in a camera-image in the article;

5 Estimated from given mirror-diameter 25.4 mm/484 pixels and aperture diameter 0.8 mm. Directions are limited to *θ* ≤ 23° to *θ* ≤ 37° (depending on *ϕ*, see Figure 21a). Light samples are cones with 2.5°–6.6° diameter;

6 Theoretical value for a complete measurement (probably never attempted). See discussion in Section 8.2.2. ;

7 Estimated from the texture resolution reported in the paper and the size of a depicted M&M candy;

8 Median head breadth and menton-crinion length of a male Caucasian;

9 Estimated using camera-images of male Caucasian faces depicted in the article;

10 The speed is given for monochromatic bidirectional reflectance samples. Possible multiple exposures for HDR combination are not considered. For oblique views the full resolution is assumed as well;

11 Based on timings including the 3D acquisition, as this cannot be separated in this approach;

12 Based on timings including the 3D acquisition, because no separate timings are available;

13 Average speed over all available measurements.
